# Arene Ru(II) Complexes with Difluorinated Ligands Act as Potential Inducers of S-Phase Arrest via the Stabilization of *c-myc* G-Quadruplex DNA

**DOI:** 10.3390/molecules27061897

**Published:** 2022-03-15

**Authors:** Liang Zeng, Chanling Yuan, Jing Shu, Jiayi Qian, Qiong Wu, Yanhua Chen, Ruzhen Wu, Xiaoming Ouyang, Yuan Li, Wenjie Mei

**Affiliations:** 1 Department of Pathology, Guangzhou Women and Children’s Medical Center, Guangzhou Medical University, Guangzhou 510623, China; zlxx03@126.com (L.Z.); lizzyklarck@126.com (Y.L.); 2 School of Pharmacy, Guangdong Pharmaceutical University, Guangzhou 510006, China; ycc2552@163.com (C.Y.); 15768980174@126.com (J.S.); qianjiayi0101@163.com (J.Q.); amandachen423@163.com (Y.C.); wrzjq123@163.com (R.W.); 3 Guangdong Province Engineering and Technology Centre for Molecular Probe and Biomedicine Imaging, Guangzhou 510006, China; wuqiongniu.1113@163.com; 4 Department of Pathology, The Second Affiliated Hospital of Guangzhou Medical University, Guangzhou 510260, China

**Keywords:** difluorinated ligands, arene Ru(II) complexes, *c-myc* G-quadruplex DNA

## Abstract

Here, a series of half-sandwich arene Ru(II) complexes with difluorinated ligands [Ru(*η*^6^-arene)(L)Cl] (**L_1_** = 2-(2,3-difluorophenyl)imidazole[4,5*f*][1,10]-phenanthroline; **L_2_** = 2-(2,4-difluorophenyl)imidazole[4,5*f*][1,10]-phenanthroline; arene = benzene, toluene, and *p*-cymene) were synthesized and characterized. Molecular docking analysis showed that these complexes bind to *c-myc* G-quadruplex DNA through either groove binding or *π*–*π* stacking, and the relative difluorinated site in the main ligand plays a role in regulating the binding mode. The binding behavior of these complexes with *c-myc* G-quadruplex DNA was evaluated using ultraviolet–visible spectroscopy, fluorescence intercalator displacement assay, fluorescence resonance energy transfer melting assay, and polymerase chain reaction. The comprehensive analysis indicated that complex **1** exhibited a better affinity and stability in relation to *c-myc* G-quadruplex DNA with a DC_50_ of 6.6 μM and Δ*T_m_* values of 13.09 °C, than other molecules. Further activity evaluation results displayed that this class of complexes can also inhibit the growth of various tumor cells, especially complexes **3** and **6**, which exhibited a better inhibitory effect against human U87 glioblastoma cells (51.61 and 23.75 μM) than other complexes, even superior to cisplatin (32.59 μM). Owing to a befitting lipophilicity associated with the high intake of drugs by tumor cells, complexes **3** and **6** had favorable lipid-water partition coefficients of −0.6615 and −0.8077, respectively. Moreover, it was found that complex **6** suppressed the proliferation of U87 cells mainly through an induced obvious S phase arrest and slight apoptosis, which may have resulted from the stabilization of *c-myc* G-quadruplex DNA to block the transcription and expression of *c-myc*. In brief, these types of arene Ru(II) complexes with difluorinated ligands can be developed as potential inducers of S-phase arrest and apoptosis through the binding and stabilization of *c-myc* G-quadruplex DNA, and could be used in clinical applications in the future.

## 1. Introduction

*c-myc* is an important proto-oncogene upregulated in many types of cancer, including cervical, colon, breast, lung, and glioblastoma [1]. Studies have shown that the G-rich sequence nuclease hypersensitivity element III1 in the promoter region of *c-myc*, which controls 80% to 90% of the transcription behavior of oncogenes, can forma G-quadruplex conformation under certain conditions and can inhibit the transcription of *c-myc* [2]. Therefore, the use of *c-myc* G-quadruplex DNA as a potential target has attracted wide attention. To date, researchers have not succeeded in developing any drug that targets *c-myc* and can be utilized in clinics. Finding small molecule inhibitors that can directly act on the *c-myc* protein has long been a major problem in international drug development because *c-myc* is an intrinsically disordered protein and promotes available drug discovery [3,4].

Ruthenium complexes exhibit a low general toxicity and accumulate in cancer cells due to their high rate of ligand exchange, the range of accessible oxidation states, and their ability to simulate iron binding to certain biomolecules [5]. As a result, ruthenium complexes are expected to be the next anticancer drugs [6]. A large number of Ru(II) complexes have been described in the literature, but only a few have significant antitumor activity. NAMI-A (ImH[*trans*-RuCl_4_(dmso)(Im)], where Im = imidazole) was the first ruthenium complex in clinical trials. The ruthenium complex KP1019 (IndH[*trans*-RuCl_4_(Ind)_2_], where Ind = indazole) has also been entered into cancer clinical trials; however, its low solubility has limited further study and it has been replaced by a better soluble sodium salt, KP1339 [7,8]. The arene Ru(II) complex [(*η^6^*-arene)Ru(X)(Y)(Z)] (XY is N,N-, N,O-, O,O- or S,O- chelating ligand, Z is a monoanionic ligand) has a high cytotoxicity in vivo and inhibits tumor cell growth [9]. The arene ligands are strongly coordinated with ruthenium; they stabilize the ruthenium in the oxidation state +2 and provide a hydrophobic side for passive transport through the cell membrane, and the chelating ligand and the leaving group (Cl) regulate the reactivity of the complex, resulting in cytotoxicity [10,11]. We have reported that arene Ru(II) complexes coordinated by phenanthroimidazole derivatives can inhibit tumor cell growth by stabilizing G-quadruplex DNA to induce tumor cell cycle arrest, DNA damage, and apoptosis [12,13,14]. Furthermore, it was found that these types of arene Ru(II) complexes can selectively bind to *c-myc* G-quadruplex DNA through the groove binding mode to inhibit the proliferation, migration, and invasion of cancer cells [15]. Recently, fluorine-containing drugs accounted for approximately 25% of the new drugs approved by the Food and Drug Administration (FDA), and their therapeutic areas are mainly antitumor, anti-infection, and cardiovascular systems. Compared with other halogens, fluorine has a unique electronic structure with the strongest electronegativity, similar to the radius of hydrogen atoms. These chemical properties make fluorine a value-added substitute for other atoms in medicinal chemistry. The introduction of fluorine atoms or fluorine-containing substituents into biologically active molecules can regulate a series of overall effects, such as modulating the physicochemical properties of the molecular scaffolds and affecting the polarity or hydrophilicity/lipophilicity, thereby changing the molecular membrane and the permeability of the blood–brain barrier. The fluorination effect can adjust metabolic stability, and the substitution of fluorine for hydrogen on the aromatic ring is an extremely effective strategy, which can significantly delay the oxidative metabolism of the given drug under the action of cytochrome P450 monooxygenase.

**Scheme 1:**
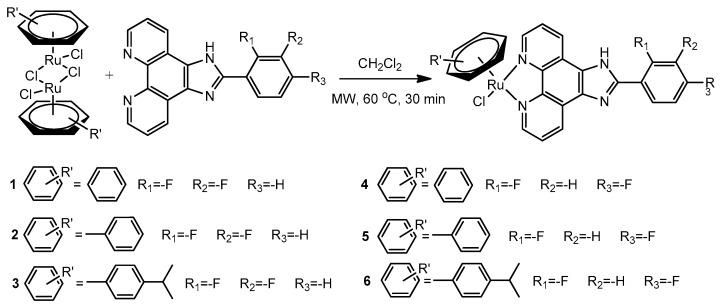
The synthesis route of arene Ru(II) complexes (**1**–**6**).

In this study, we synthesized a class of arene Ru(II) complexes with difluorinated ligands to produce a range of overall positive effects through the arene ligands and the introduction of fluorine atoms (Scheme 1). The affinity, stability, and selectivity of the arene Ru(II) complexes to *c-myc* G-quadruplex DNA were evaluated through electronic spectra, fluorescence spectra, fluorescence resonance energy transfer (FRET), and molecular docking. The results illustrated that the arene Ru(II) complexes can inhibit the proliferation of tumor cells by S-phase arrest via the binding and stabilization of *c-myc* G-quadruplex DNA.

## 2. Results and Discussion

### 2.1. Synthesis and Characterizations

All arene Ru(II) complexes with difluorinated ligands were prepared in reasonable yields by reacting Ru(II) dinuclear [(*η*^6^-benzene)RuCl_2_]_2_, [(*η*^6^-toluene)RuCl_2_]_2_ and [(*η*^6^-*p*-cymene)RuCl_2_]_2_ with the corresponding ligand (L) in CH_2_Cl_2_ at 60 °C under microwave irradiation for 30 min in a Pyrex vessel (Scheme 1). Moreover, the molecular structures were characterized through ESI–MS, (Supplementary Materials, Figures S1–S8), elemental analysis, and ^1^H NMR and ^13^C NMR (Figures S9–S16). As shown in Figure S11, the peaks at *δ*~9.98, 9.39, and 8.21 of complex **1** were ascribed to protons in the phenanthroline ring **L_1_** of H_1_, H_3_, and H_2_, respectively. The peaks of *δ*~8.15, 7.67, and 7.47 were attributed to H_6_, H_5_, and H_4_ in the phenyl ring of phenanthroline coupled with each other, respectively. The peak of *δ*~6.34 was attributed to the resonance of the corresponding H_7_ protons in the C_6_H_6_ ring. For **2**, the peaks at *δ*~6.10, 6.46, and 5.91 were assigned to H_b_, H_c_, and H_d_ in the toluene ring of the auxiliary ligand, respectively. The peak at *δ*~2.21 was assigned to H_a_ in the toluene ring. For **3**, the peaks at *δ*~2.63 and 0.93 were attributed to H_d_ and H_e_ in the *p*-cymene ring of the auxiliary ligand, respectively. The peaks at *δ*~2.22 were attributed to H_a_ in the *p*-cymene ring, and the peaks at *δ*~6.37 and 6.13 were assigned to H_c_ and H_d_ in the *p*-cymene ring, respectively. The ^1^H NMR of complexes **4**, **5**, and **6** can be analyzed in the same manner. The phenanthroimidazole ligands in arene Ru(II) complexes are coordinated to Ru(II) in a bidentate fashion through the N atom of 1,10-Phenanthroline monohydrate in the neutral form. The available data from the literature have also shown that phenanthroimidazole derivatives act as bidentate chelating ligands [16]. Monodentate chloride acts as a leaving group in the other positions.

The role of halogen bonding (X-bonding) in drug development, in which a halogen atom exhibits a highly directional attraction to an electron donor to benefit from interactions with drug targets, has attracted notable attention for a long time [17]. Previous studies found that the types of arene Ru(II) complexes containing different halogen substitutes exhibited greater anti-tumor activity and a stronger G-quadruplex DNA binding ability than when no halogen substitutes were used, especially for F-substitutes and Cl-substitutes [13]. In order to explore the complexes with a high effectiveness and a low toxicity, we introduced the F element in the structure of the arene Ru(II) complexes to promote their biological effects.

In this study, the phenyl ring that is directly connected to ruthenium in the original parent structure was modified, and difluorophenyl was introduced into the ligand. It was mainly the substituents of the compound that were changed. According to the structural analysis of this type of arene Ru(II) complex, the single crystal structures of these complexes in this study have pseudo-octahedral “piano-stool” structures with the neutral arene ligand occupying three coordination positions (the “seat”) and difluorinated ligand and monodentate chloride occupying the other positions (the “legs”)

### 2.2. G4 DNA Binding Behaviors

#### 2.2.1. Molecular Docking

**Figure 1:**
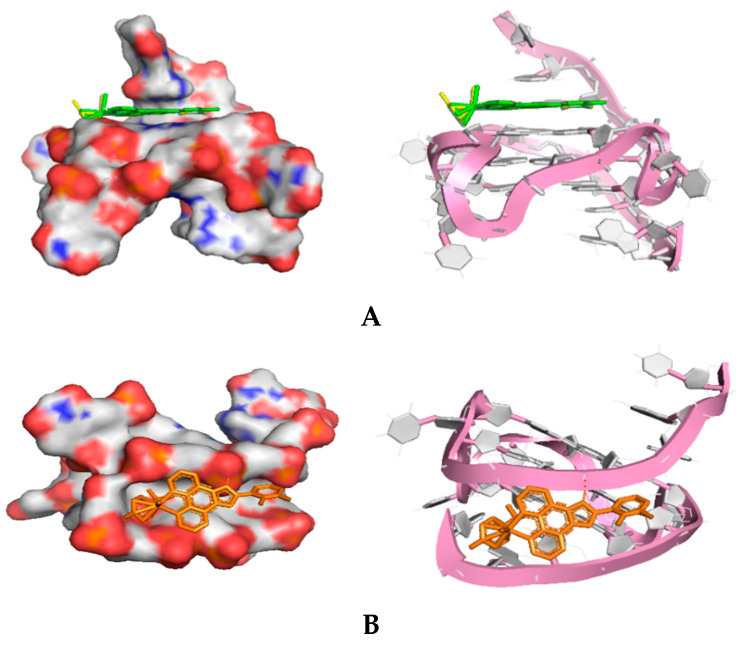

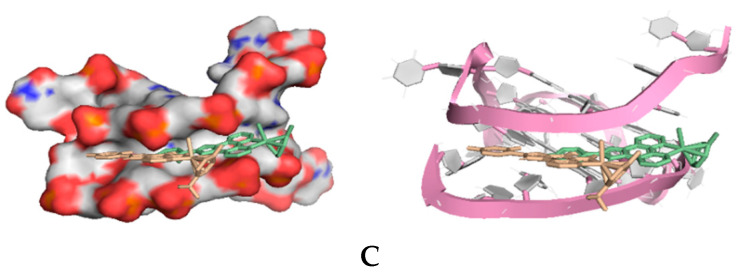
Binding mode and site of the arene Ru(II) complexes that interacted with *c-myc* G-quadruplex DNA analyzed by molecular docking. Left (G-quadruplex is rendered with the hydrophobic surface of molecular structure), Right (G-quartets are displayed in a stick mode) (**A**) **1** (green), **2** (yellow) and **4** (cyan); (**B**) **3** (orange); (**C**) **5** (pale green) and **6** (wheat).

The interaction of these difluorinated arene Ru(II) complexes with *c-myc* G-quadruplex DNA was investigated through molecular docking. The G-quadruplex conformation formed by G-rich *c-myc* promoter sequences (PDB ID 2L7V) from the PDB database was used in docking studies with the ligands using AutoDock 4.2 software, and PyMOL was used for rendering images. The analysis results show that the molecular structure of complexes **1** and **2** mainly stacked onto the external G-quartet plane formed by the G7, G11, G16, and G20 base pairs of *c-myc* G4-DNA through a large aromatic plane (Ligand **L_1_**) G4-DNA via π–π stacking (Figure 1A), and the binding energy was approximately −7.09 and −7.17 kcal/mol, which directly influenced the stability of G-quartet; the molecular structures of complexes **3** and **4** inserted the groove constructed by G7–G9 and G21–G25 of G4-DNA through the head (the phenyl ring coordinated with ruthenium) and tail (the terminal F-modified phenyl ring in Ligand **L_1_** or **L_2_**) to impact the loop structural features, with a binding energy of −7.06 and −6.99 kcal/mol (Figure 1B); however, the molecular structures of complexes **5** and **6** interacted with the groove of G4-DNA just through the tail (the terminal F-modified phenyl ring in Ligand **L_2_**), which interfered with the loops to a certain degree (Figure 1C). Furthermore, molecular docking analysis also indicated that the complex without an F atom displayed a certain degree of affinity to *c-myc* G-quadruplex DNA through the groove binding mode with a binding energy of −7.13 kcal/mol, but with the introduction of the F atom in the terminal benzene ring at the ortho-position and para-position, the complexes bound to *c-myc* G-quadruplex DNA through π–π stacking with binding energies of −7.23 and 7.19 kcal/mol, respectively (Figure S21). These results suggest that the introduction of an F atom can adjust the interaction mode of this class of complex with *c-myc* G-quadruplex DNA with little change in the binding energy. The above results are in agreement with the FRET melting assay, which showed that the weaker binding ability resulted in *c-myc* G4-DNA with a lower stability. Comprehensive analysis results indicated that the space hindrance of the coligand (benzene unit coordinated with Ru atom) plays a key role in determining the binding site of these complexes in *c-myc* G-quadruplex DNA, and the introduction of an F atom in the terminal benzene ring can adjust the interaction mode of a complex with *c-myc* G-quadruplex DNA.

#### 2.2.2. UV–Vis Spectra

**Figure 2:**
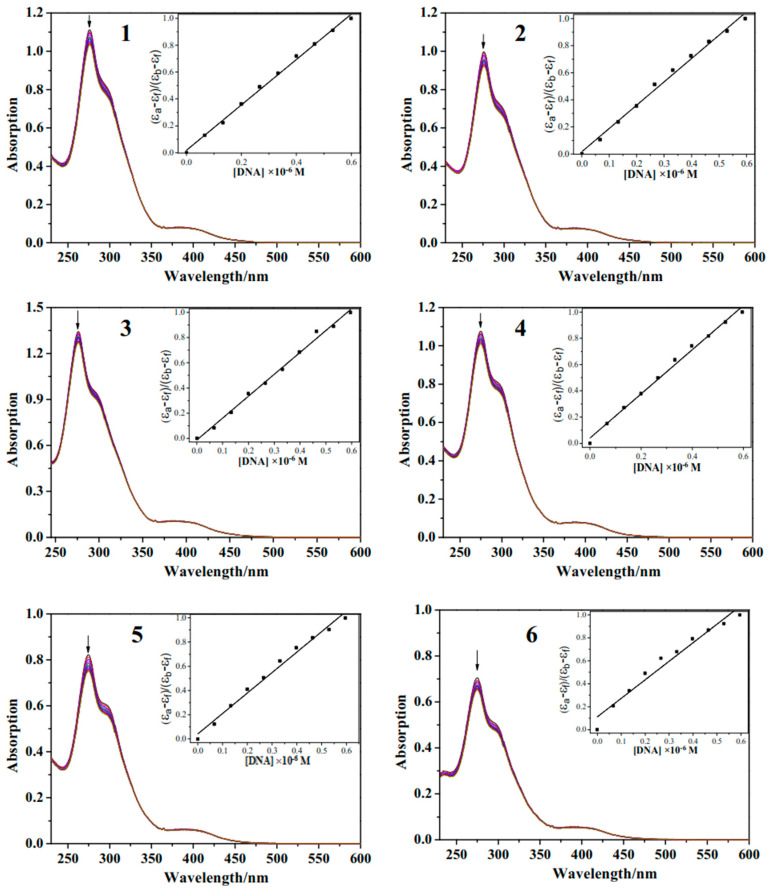
Representative UV–vis absorption titrations of arene Ru(II) complexes upon the addition of *c-myc* G-quadruplex DNA in a 10 mM Tris-HCl buffer (pH 7.4) containing 100 mM KCl. The arrow shows the absorbance change with the increase in *c-myc* G-quadruplex DNA concentrations.

Electron spectroscopy is one of the most widely used techniques to evaluate the binding ability between metal complexes and DNA. UV–vis spectra titration experiments were conducted to further clarify the binding behavior of these complexes with *c-myc* G-quadruplex DNA. Figure 2 shows the UV–vis spectra of Ru(II) complexes with and without *c-myc* G-quadruplex DNA in a Tris-HCl-KCl buffer. The electronic spectra of these arene Ru(II) complexes showed a characteristic absorption at approximately 275 nm, which can be attributed to the intraligand (IL) charge transfer, and the MLCT peak observed at about 386 nm can be attributed to metal-to-ligand charge transfer (MLCT) absorption. Upon the addition of *c-myc* G-quadruplex DNA, it was observed that the absorption peak of the compound decreased and there was an obvious hypochromic effect. The calculated hypochromic rates for **1**, **2**, **3**, **4**, **5,** and **6** at the IL absorption band were approximately 7.13%, 7.55%, 5.46%, 6.15%, 8.83%, and 7.50%, respectively. The intrinsic equilibrium binding constants (*K*_b_) calculated for **1**, **2**, **3**, **4**, **5,** and **6** with *c-myc* G-quadruplex were approximately 67.5, 110, 134, 48.3, 41.1, and 14.6 × 10^6^ M^−1^, respectively. Moreover, the complex without the F atom exhibited a weak affinity to *c-myc* G-quadruplex DNA, and there were tiny changes in the hypochromic effect (3.5%) with the binding energy of 1.5 × 10^5^ M^−1^. However, with the introduction of the F atom in the terminal benzene ring at the para-position, the complex displayed an obvious hypochromic effect (7.1%) with a binding energy of 6.4 × 10^5^ M^−1^ (Figure S22). These data are comparable with that of arene ruthenium complexes [(*η*^6^-C_6_H_6_)RuCl(pmpzdpm)] (4.29 × 10^5^ M^−1^) and [(*η*^6^-C_10_H_14_)RuCl(pmpzdpm)] (1.79 × 10^6^ M^−1^) (pmpzdpm = 5-(2-pyrimidylpiperazine)phenyldipyrromethane) [18] and [Ru(bpy)_2_ASC]^2+^ (2.78 × 10^6^ M^−1^) (ACS = ascididemin) [19]. 

Moreover, it was found that the complex containing one F-substituent exhibited a stronger *c-myc* G-quadruplex DNA (G4 DNA) affinity than the complex without an F-substituent. Then, compared with the complexes with and without an F-substituent, the introduction of two F atoms at the ortho and para positions into the molecular structure led to a better *c-myc* G4 DNA binding ability [15]. These data suggest that the introduction of an F atom can promote interactions between the arene Ru(II) complex and *c-myc* G-quadruplex DNA.

#### 2.2.3. Fluorescence Intercalator Displacement (FID) Assay

An FID assay was performed to further illustrate the interactions of these arene Ru(II) complexes with *c-myc* G-quadruplex DNA. As a sensitive fluorescence probe for the detection of DNA structure, thiazole orange (TO) is highly fluorescent upon interaction with G-quadruplex DNA (~500- to 3000-fold exaltation) and is totally quenched when free in solution (quantum yield (*Ø*) = 2 × 10^−4^). The displacement ability of the ruthenium complexes can be easily monitored by the decrease in TO fluorescence (*λ_max_* = 530 nm) upon selective excitation at 501 nm [20]. The binding affinity of these complexes with *c-myc* G-quadruplex DNA is represented by DC_50_, where the DC_50_ value represents the concentration of the arene Ru(II) complexes required to reduce the fluorescence intensity by 50% [21]. A low DC_50_ indicates that the arene Ru(II) complexes have a strong binding ability to G-quadruplex DNA [22].

**Figure 3:**
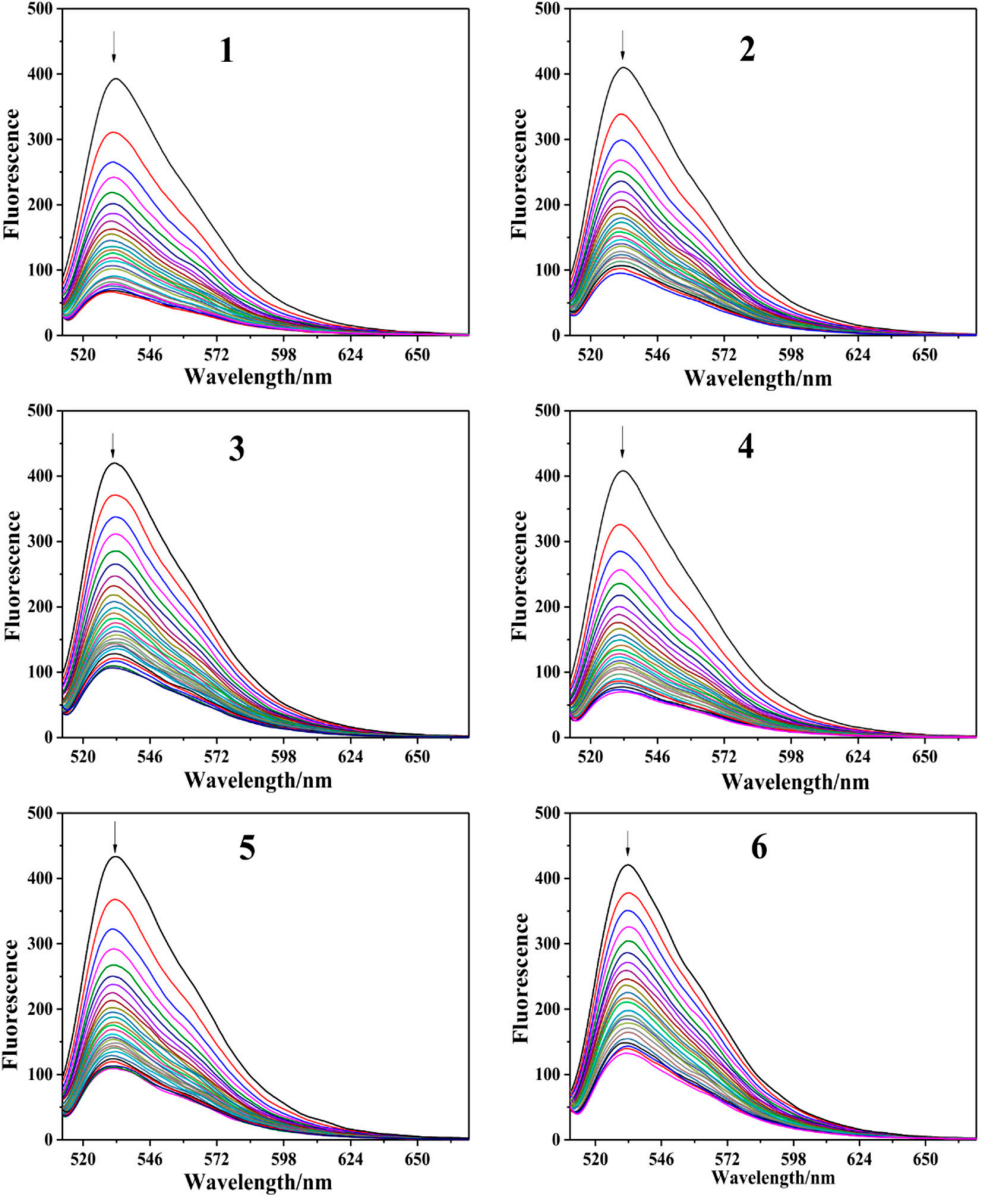
Thiazole orange (TO) displacement by arene Ru(II) complexes from *c-myc* G-quadruplex-TO adduct. The fluorescence spectra of the TO/G-quadruplex are obtained in a buffer containing 10 mM Tris-HCl, 100 mM KCl, pH 7.4 with increasing concentrations of arene Ru(II) complexes. [TO] = 1 μM, [Ru] = 0–45 μM, [*c-myc*] = 0.50 μM, λex = 501 nm.

**Figure 4:**
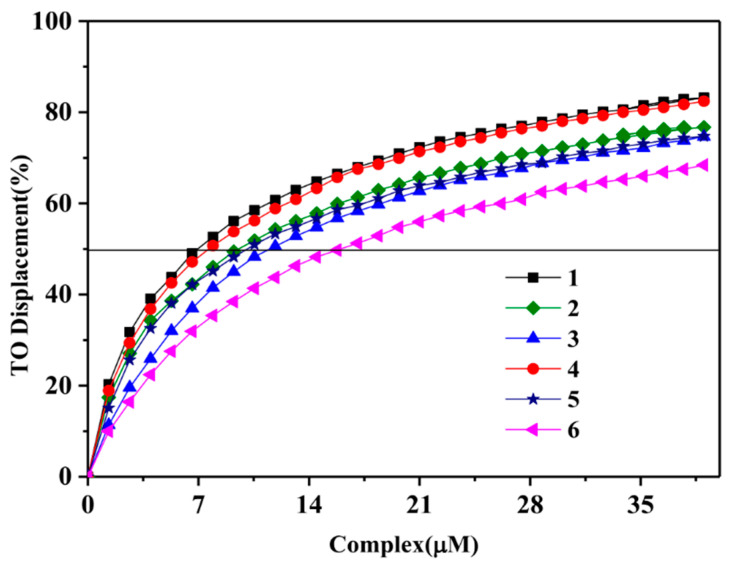
The DC_50_ values of Ru(II) complexes. The relative TO displacement for *c-myc* G-quadruplex substrates as a function of increasing concentration of the specified complexes is shown.

As shown in Figure 3, TO-DNA emits a strong fluorescence at 512–680 nm when it is excited at 501 nm. With the increase in arene Ru(II) complexes to the TO-DNA, the fluorescence intensity gradually decreases following the increase in arene Ru(II) complexes. This condition is due to the competitive relationship between the arene Ru(II) complexes and the TO bound in the TO-DNA. Table 1 shows the DC_50_ values obtained from all FID analyses. As shown in Figure 4, the DC_50_ values of **1**, **2**, **3**, **4**, **5,** and **6** were 6.6, 9.93, 10.26, 7.8, 10.8, and 15.6 µM respectively. The above results indicate that these arene Ru(II) complexes have some capacity to bind to *c-myc* G-quadruplex DNA, especially complex **1,** which had the strongest capacity.

#### 2.2.4. Fluorescence Resonance Energy Transfer (FRET) Melting Assay

**Figure 5:**
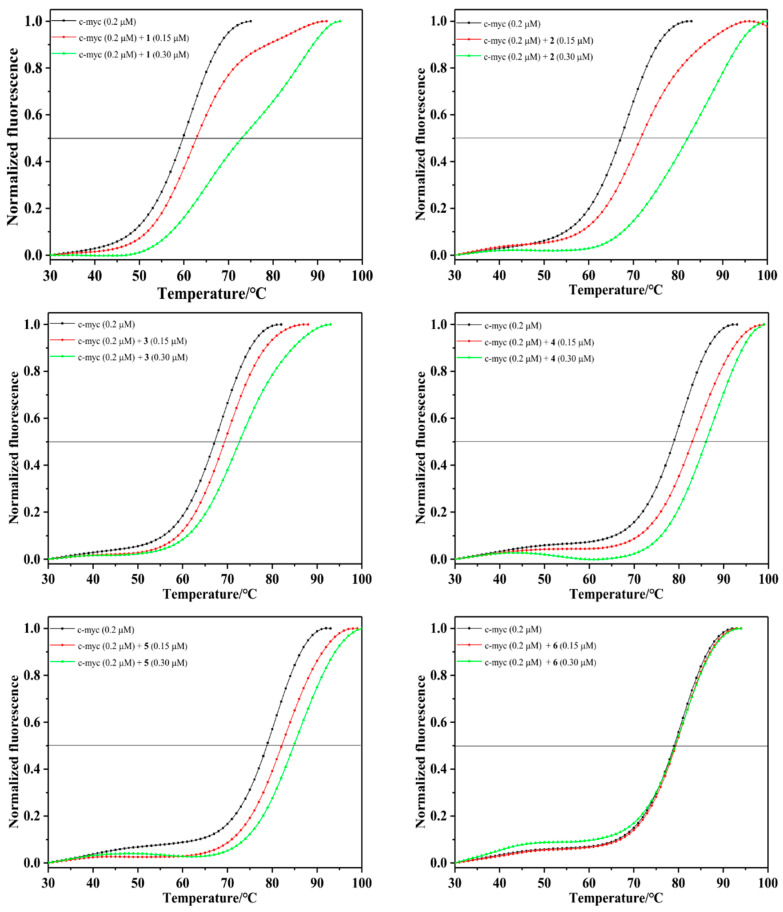
Normalized FRET melting curves of *c-myc* G-quadruplex DNA (0.2 μM) in a K^+^ buffer, with increasing concentrations (0–0.3 μM) of Ru(II) complexes (**1**–**6**) measured by real-time polymerase chain reaction PCR system.

**Figure 6:**
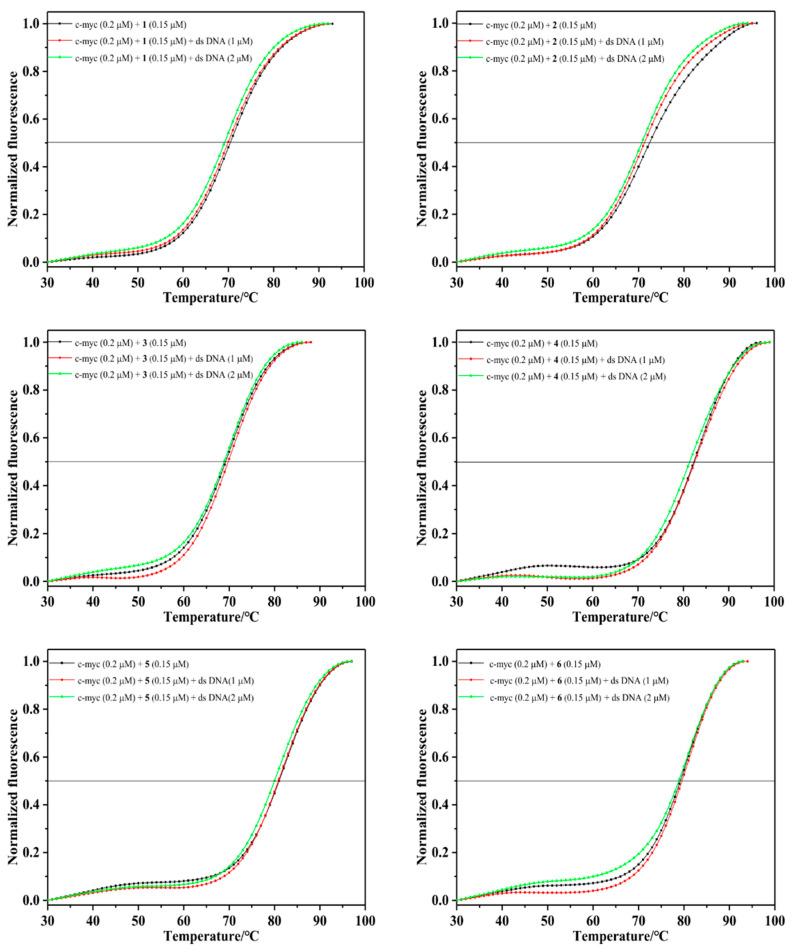
Competitive FRET-melting curves of *c-myc* (0.20 μM) with 0.15 μM of Ru(II) complexes (**1**–**6**) and a duplex competitor ds26 (1–2 μM) in a buffer containing 100 mM KCl.

The data from ultraviolet and FID experiments indicate that arene Ru(II) complexes have a certain binding ability with G-quadruplex DNA. To further verify these results, we studied the stability of arene Ru(II) complexes to the structure of G-quadruplex DNA through the FRET experiment. As shown in Figure 5, the melting point of *c-myc* enhances with the increase in the concentration of the complexes, indicating that the complexes bound to *c-myc* can improve the stability of G-quadruplex DNA. The Δ*T_m_* values of **1**, **2**, **3**, **4**, **5,** and **6** were 13.09 °C, 15.05 °C, 5.07 °C, 7.23 °C, 6.03 °C, and 0.4 °C, respectively. The six arene Ru(II) complexes, especially complex **2**, were stable in relation to *c-myc* G-quadruplex DNA,. The selectivity of the arene Ru(II) complexes to G-quadruplex DNA and ds DNA was explored through a competitive FRET experiment. In the experiment, the selectivity of the six arene Ru(II) complexes to G-quadruplex DNA was better than that of ds DNA. The competition FRET melting assay was conducted to show the ∆*Tm* change for 0.15 µM of the complex with 0.2 µM *c-myc* by adding different concentrations of the double-stranded DNA (Figure 6). ∆*Tm* (0.4–1.2 °C) showed minimal change when different concentrations of ds DNA were added, indicating that the arene Ru(II) complexes were highly selective to G-quadruplex DNA compared with ds DNA. These results suggest that the arene Ru(II) complexes that bind to *c-myc* G4 DNA may be beneficial to the stability of the G-quadruplex conformation.

### 2.3. Biological Studies

The antiproliferative activities of synthesized arene Ru(II) complexes against various human cancer cell lines were evaluated through MTT assay. All these complexes exhibited a certain inhibition in relation to lung cancer A549 tumor cells after 72 h of treatment, and **6** had a better antitumor activity to U87. Under the same conditions, arene Ru(II) complexes typically have no effect on MCF-7 and HepG2 cells, suggesting that arene Ru(II) complexes are selective for specific cells.

In order to further evaluate the stability of complex **6** in a buffer solution, the UV absorption value of complex **6** on the first day was compared with the absorption peak after three days of storage (Figure S18). The experimental results showed that the UV–vis spectra of the compound after three days in the buffer solution were consistent with that of the first day of preparation. There was some deviation but this was permissible.

The antitumor activities of the arene Ru(II) complexes (**1**–**6**) were evaluated against human breast cancer MCF-7 cells, human lung cancer A549 cells, human liver carcinoma HepG2 cells, and human glioblastoma U87 cells using an MTT assay. The inhibitory effects (IC_50_) of these arene Ru(II) complexes (**1**–**6**), the ligand (**L_1_** and **L_2_**), and cis-platin against the growth of various cell lines after 72 h of treatment are listed in Table 2. The results show that complex 6, which was the most active, can effectively inhibit the growth of U87 cells with an IC_50_ value of 23.75 μM.

These complexes exhibited a significant inhibitory effect on the selected tumor cell lines after 72 h of treatment. For ligands L_1_ and L_2_, these two compounds exhibited extremely excellent anti-tumor activity against different cancer cells, especially for HepG2 cells with IC_50_ values of 0.29 μM and 0.12 μM, respectively. However, these two compounds also exhibited obvious toxicity in relation to human normal liver cells with IC_50_ values of 0.34 μM and 0.18 μM, respectively. Complexes **3** and **6** (coordinated with *p*-cymene) exhibited greater inhibition than **1**, **4** (coordinated with benzene) and **2**, **5** (coordinated with toluene). The results indicate that the arene Ru(II) complexes coordinated by *p*-cymene displayed excellent inhibitory effects against lung cancer A549 cells and glioblastoma U87 cells. Notably, **6** exhibited the best antiproliferative activity against the U87 cells (IC_50_, 23.75 μM), which was better than cis-platin with an IC_50_ value of 32.59 μM. The substituent effects (steric effect, electronic effect, and position of substituted groups) have a major effect on the biological activities of the drugs [23,24]. Compared with the IC_50_ of these arene Ru(II) complexes, the IC_50_ values of **4** and **6** (coordinated with *p*-cymene) indicate that their anticancer activity against the A549 and U87 cells was higher than the other complexes (coordinated with benzene or toluene) under the same conditions. These results suggest that a steric factor is critical for the antitumor activities of these complexes.

Subtle changes in the structural modification would reflect a different aqueous solubility as well as an acceptable degree of lipophilicity and hydrophilicity, which is necessary to allow the complex to cross the cell membrane and achieve the appropriate plasma and intracellular concentrations. The anticancer potential is in direct correlation with befitting lipid–water partition coefficients [25]. The umbrella-shaped arene Ru(II) complexes exhibited a higher order of logP*o*/*w* due to the lipophilic nature of their isopropyl group; therefore, **3** and **6** had a better biological activity than other complexes, especially **6**, which exhibited a great inhibitory effect against glioblastoma U87 cells.

### 2.4. Joint Action of S-Phase Arrest and Apoptosis

**Figure 7:**
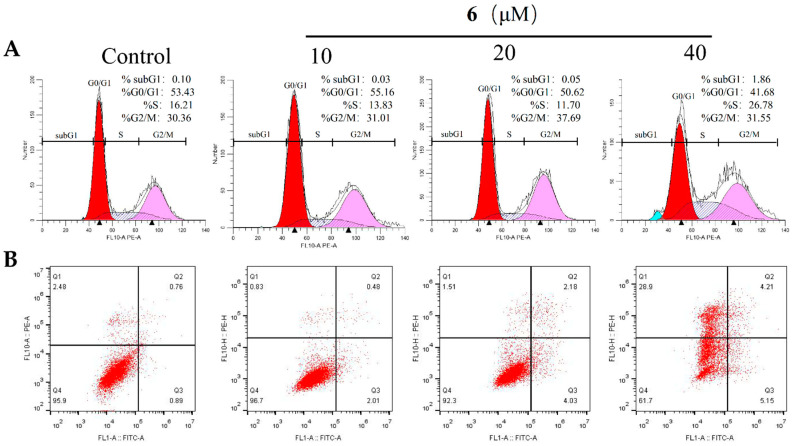
S-phase arrest (**A**) and apoptosis (**B**) of U87 cells induced by **6** for 72 h tested by flow cytometric analysis.

The cytomorphology evaluation was conducted in U87 cells because **6** showed a low IC_50_ concentration in the cytotoxicity assay. After 72 h of incubation with the additional concentration of **6**, the proportion of cells at the S-phase increased significantly, accompanied by a decrease in the percentage of cells at the G0/G1 phase (Figure 7A). Moreover, an obvious subG1 signal at the concentration of 40 μM was observed. Further study showed that this complex also induced U87 cells to apoptosis, which was in agreement with the cell cycle analysis results. In particular, at 40 μM an increasing ratio of apoptosis and necrosis was observed (Figure 7B). These results suggest **6** inhibited cell proliferation mainly through inducing the joint action of S-phase arrest, cell apoptosis, and necrosis.

## 3. Experimental Section

### 3.1. Materials and Methods

#### 3.1.1. Chemicals

Ruthenium(III) chloride hydrate was obtained from Mitsuwa Chemicals. 1,10-Phenanthroline monohydrate, 1,3-cyclohexadiene, 1-methyl-1,4-cyclohexadiene, and 1,3-cyclohexadiene were purchased from Aldrich. All other starting materials and solvents were obtained from commercial vendors and used without any further purification. The *c-myc* G-quadruplex DNA (5′-TGGGGAGGGTGGGGAGGGTGGGGAAGG-3′) was purchased from General Biol (Anhui) Co., Ltd. Chuzhou, China The Tris-KCl buffer solution consisted of 10 mM Tris and 100 mM KCl, and the pH value was adjusted to 7.4 by HCl solution, which was applied to the UV–vis titration, FID, and FRET experiments.

#### 3.1.2. Instruments

The arene Ru(II) complexes were synthesized using a microwave reactor (Anton Paar GmbH; Shanghai, China, monowave 300). ESI–MS spectra were obtained in methanol on an Agilent 1100 ESI–MS system (Agilent, Palo Alto, CA, USA) operating at room temperature. The ^1^H NMR and ^13^C NMR spectra were recorded in DMSO-*d*^6^ with a Bruker DRX2500 spectrometer operating at room temperature. The UV–vis spectra were recorded with a Shimadzu UV-2550 spectrophotometer. The FID assay was recorded with an RF-5301 fluorescence spectrophotometer. The FRET assay was recorded with a Bio-Rad real-time PCR (CFX96 Touch).

#### 3.1.3. Cell Lines and Culture

Human cancer cell lines, including breast cancer (MCF-7), lung cancer (A549), liver cancer (HepG2), and glioblastoma (U87) were purchased from American Type Culture Collection (Manassas, VA, USA). The cell lines of MCF-7, HepG2, A549, and U87 were maintained in Dulbecco’s Modified Eagle Medium (DMEM) with fetal bovine serum (10%), penicillin (100 units/mL), and streptomycin (50 units/mL) at 37 °C in a CO_2_ incubator (95% relative humidity, 5% CO_2_). Cells were maintained in DMEM with horse serum (10%), penicillin (100 units/mL), streptomycin (50 units/mL), EGF (20 ng/mL), hydrocortisone (0.5 μg/mL), cholera toxin (100 ng/mL), and insulin (10 μg/mL) at 37 °C in a CO_2_ incubator (95% relative humidity, 5% CO_2_).

#### 3.1.4. MTT Assay

All arene Ru(II) complexes were dissolved in DMSO with stock solution at 10 mg/mL. Cell viability was determined by measuring the ability of cells to transform MTT to a purple formazan dye. Cells were seeded in 96-well tissue culture plates (3 × 10^3^ cells per well) for 24 h. The cells were then incubated with the tested compounds at different concentrations for 72 h. After incubation, 20 μL per well of MTT solution (5 mg/mL PBS) was added and incubated for 5 h. The medium was aspirated and replaced with DMSO (150 μL per well) to dissolve the formazan salt. The absorbance intensity, which reflects the cell growth condition, was measured at 570 nm using a microplate spectrophotometer (Versamax).

#### 3.1.5. n-Octanol-Water Partition Coefficient (logPo/w)

Experimental 1-octanol/water partition coefficients (Po/w) were determined with the slow-stirring method [26], and 1-octanol and Mili-Q water were mutually saturated prior to their use. The arene Ru(II) complex of a known concentration in mixture (water saturated with 1-octanol and 1-octanol saturated with water) was shaken for 24 h on an oscillator. The solution was centrifuged for 5 min at 2500 rpm to allow the phase separation. The amount of ruthenium present in the saturated aqueous solution and saturated 1-octanol solution was measured through UV–vis spectroscopy [27], Here, the 1-octanol/water partition coefficient (logP*o*/*w*) is defined as the logarithm of the ratio between the molar concentration of complex in the octanol-rich phase(*Co*) and the molar concentration of complex in the water-rich phase(*C*):

logP*o*/*w* = logC*o*/C (1)

#### 3.1.6. UV–Vis Titration

The intramolecular *c-myc* G-quadruplex DNA structure was prepared by incubating Pu22 in a 10 mM Tris-HCl buffer (pH 7.4) containing 100 mM KCl, which was heated to 95 °C for 5 min, cooled to room temperature overnight, and stored at 4 °C for 24 h [4]. The binding parameters of the arene Ru(II) complexes and *c-myc* G-quadruplex DNA were measured at room temperature using the UV–vis absorption spectra (Shimadzu UV-2550 spectrophotometer). This process was performed by keeping the concentration of arene ruthenium complexes the same (20 μM), changing the concentration of *c-myc* G-quadruplex DNA (0–60 μM), and performing electron absorption titration in the range of 200–800 nm. The intrinsic binding constant (K_b_) was calculated using the following:

[DNA]/(εa − εf) = [DNA]/(εb − εf) + 1/[Kb(εb − εf)](2)

In the formula, εa, εf, and εb are the apparent coefficients, and the free and fully bound forms of the metal complex extinction coefficients, respectively. A plot of [DNA]/(εa − εf) versus [DNA] gave a slope of 1/(εb − εf) and a Y-intercept equal to 1/Kb (εb − εf). K_b_ is the ratio of the slope to the intercept.

#### 3.1.7. FID Assay

The binding parameters of the arene Ru(II) complexes and *c-myc* G-quadruplex DNA were measured at room temperature using an FID assay. To the annealed *c-myc* G-quadruplex DNA (0.5 μM), 1.0 μM of thiazole orange dye was added and allowed to equilibrate for 0.5 h. The emission spectrum was recorded in the range of 512–680 nm with an excitation wavelength of 501 nm. The arene Ru(II) complexes (0–60 μM) were added to the *c-myc* G-quadruplex DNA–TO complex, and the emission spectra were recorded for each addition after 2 min of equilibration. The fluorescence area under each curve was calculated and converted to the percentage of the TO displacement value using the equation: Percentage of TO displacement = 100 − [(*F*/*F*_0_) × 100], where *F*_0_ = fluorescence area without a ligand, and *F* = fluorescence area in the presence of a ligand [28]. The percentage of TO displacement was plotted against the concentration of ligands, and the DC_50_ values were determined. All spectra were analyzed using Origin 9.0 software.

#### 3.1.8. FRET Melting and Competitive FRET Assays

The fluorescent-labeled oligonucleotide, *c-myc* G-quadruplex DNA (5′-FAM TGGGGAGGGTGGGGAGGGTGGGGAAGG-TAMRA-3′, FAM: carboxyfluorescein, TAMRA: 6-carboxytetra methylrhodamine) used as the FRET probe was diluted in 10 mM Tris-HCl (pH 7.4), 100 mM KCl, and 10 mM Na_3_AsO_4_ and then annealed by heating to 90 °C for 5 min, followed by a slow cooling to room temperature. Fluorescence melting curves were determined with a Bio-Rad real-time PCR detection system using a total reaction volume of 25 mL, with labeled oligonucleotide (0.20 μM) and different concentrations of the complexes (0.15 μM and 0.30 μM) in a Tris-HCl KCl buffer. The fluorescence-based melting analysis competition experiment showed the compound’s selectivity to *c-myc* G-quadruplex DNA. The experimental method was similar to the FRET melting assay but with a slight modification by adding different concentrations of double-stranded DNA (1 μM and 2 μM) as competitors. Final analysis of the data was conducted by using Origin 9.0 (Origin Lab Corp., Northampton, MA, USA).

#### 3.1.9. Molecular Docking

A molecular docking study of Ru(II) complexes was performed using the Lamarckian genetic algorithm local search method with AutoDock4.2. The spatial structures of six arene Ru(II) complexes were optimized using the Amsterdam Density Functional (ADF) 2019.104 suite program with the generalized gradient approximation (GGA): BP86 level of theory and the Mopac method, and the optimized structures were used to create the initial PDB structures using the Mercury software. This process was performed to find the most favorable orientation of these Ru(II) complexes with the *c-myc* G-quadruplex DNA under the ionosphere of potassium ion in vacuum. The crystallographic structure of *c-myc* G4-DNA was downloaded from the Protein Data Bank (PDB ID: 2L7V). Only chain A was maintained by removing other subunits. AutoDock tools were utilized to assign the Gasteiger charge and other parameters. The binding site was defined using a grid of 126 × 126 × 126 points each with a grid space of 0.375 Å centered at coordinates *x* = 2.579, *y* = −0.627, and z = −4.749. The precalculated binding affinity of each ligand’s atom type was prepared using Autogrid. The parameters of the Lamarckian genetic algorithm were as follows: 10 runs, elitism of 1, mutation rate of 0.02, population size of 150, and a crossover rate of 0.80. AutoDock 4.2 was utilized for the molecular docking simulation. Fifty separate dockings were performed with maximum of energy evaluations to 2.5 × 10^7^. The conformation corresponding to the most cluster members and the lowest binding free energy was selected as the most probable binding conformation. PyMOL was used for rendering images.

#### 3.1.10. Flow Cytometric Analysis

The apoptosis rate was analyzed through flow cytometry, as previously described. Treated or untreated cells were trypsinized, washed with PBS, and then fixed in 75% ethanol overnight at 20 °C. The fixed cells were washed with PBS and stained with propidium iodide for 4 h in the dark. The above described cells were analyzed with an Epics XL-MCL flow cytometer (Beckman Coulter, Miami, FL, USA).

### 3.2. Synthesis

#### 3.2.1. Synthesis of 2-(2,3-Di-fluorophenyl) Imidazole [4,5f][1,10]-Phenanthroline (**L_1_**)

Phenanthroimidazole derivatives were synthesized in accordance with the procedure in literature [29] with some modifications. Phenanthroline 5,6-dione (315 mg, 1.50 mmol), 2,3-difluorobenzaldehyde (320 mg, 2.25 mmol), ammonium acetate (4.0 g, 51.9 mmol), and acetic acid (17 mL) were added to a 30 mL quartz reaction tube and irradiated by microwaves for 20 min at 100 °C. After the reaction, the mixture was diluted with 100 mL water and adjusted to pH = 7 with ammonia water. The solution was filtered and dried in a vacuum to obtain a yellow product, which was purified by silica gel column chromatography. ESI-MS (in CH_3_CH_2_OH, *m*/*z*): 332.9 [M + H]^+^, 354.9 [M + Na]^+^, 687.0 [2M + Na]^2+^. ^1^H NMR (600 MHz, DMSO-*d*_6_) δ 9.05 (dd, *J* = 4.3, 1.7 Hz, 2H), 8.96 (dd, *J* = 8.0, 1.3 Hz, 2H), 8.01 (dd, *J* = 7.8, 6.4 Hz, 1H), 7.83 (dd, *J* = 8.1, 4.3 Hz, 2H), 7.65–7.59 (m, 1H), 7.45 (ddd, *J* = 12.3, 7.2, 4.7 Hz, 1H). ^13^C NMR (151 MHz, DMSO-*d*_6_) δ 148.5 (s), 146.9 (s), 146.8 (s), 145.3 (s), 144.2 (s), 130.3 (s), 123.8 (s), 121.0 (s), 120.9 (s), 118.9 (s), 118.8 (s).

#### 3.2.2. Synthesis of 2-(2,4-Di-fluorophenyl) Imidazole[4,5f][1,10]-Phenanthroline (**L_2_**)

2-(2,4-di-fluorophenyl) imidazole[4,5*f*][1,10]-phenanthroline was prepared using the method described above, but with 2,4-difluorobenzaldehyde (320 mg, 2.25 mmol). ESI-MS (in CH_3_CH_2_OH, *m*/*z*):332.9 [M + H]^+^, 354.9 [M + Na]^+^, 687.0 [2M + Na]^2+^. ^1^H NMR (600 MHz, DMSO-*d*_6_) δ 9.04 (dd, *J* = 4.3, 1.7 Hz, 2H), 8.97 (d, *J* = 7.8 Hz, 2H), 8.26 (td, *J* = 8.7, 6.6 Hz, 1H), 7.82 (dd, *J* = 8.1, 4.3 Hz, 2H), 7.57 (ddd, *J* = 11.4, 9.3, 2.5 Hz, 1H), 7.36 (td, *J* = 8.4, 2.3 Hz, 1H). ^13^C NMR (151 MHz, DMSO-*d*_6_) δ 148.4 (s), 145.6 (s), 144.1 (s), 132.4 (s), 132.3 (s), 130.3 (s), 123.8 (s), 115.8 (s), 115.7 (s), 113.1 (s), 112.9 (s).

#### 3.2.3. Synthesis of [(*η*^6^-Benzene)Ru(L_1_)Cl]Cl (**1**)

The arene Ru(II) complex **1** was synthesized in accordance with the literature [15], but with some modifications. A mixture of [(*η*^6^-benzene) RuCl_2_]_2_ (0.035 mmol, 17.5 mg), L_1_ (0.07 mmol, 22.54 mg), and dichloromethane (7 mL) was dissolved in a Pyrex vessel under the protection of N_2_ atmosphere and then heated for 30 min under microwave irradiation at 60 °C. After the solvent was evaporated, the mixture was dissolved in methanol and filtered to obtain a yellow crude product. ESI-MS (in MeOH, *m*/*z*): 546.0057 ([M − Cl]^+^); 1093.00 ([2M − 2Cl − H]^+^). This class of complexes are freely soluble in DMSO, DMF and have moderate to good solubility in H_2_O, MeOH, EtOH and CH_3_CN. Calculated forC_25_H_18_O N_4_Cl_2_F_2_Ru (%):C 50.01, H 3.02, N 9.33; Found (%): C 49.48, H 2.81, N 9.14. (One molecule containing1Cl^−^, 1 H_2_O). ^1^H NMR (500 MHz, DMSO-*d*_6_) δ 9.98 (dd, *J* = 5.2, 0.9 Hz, 2H), 9.39 (s, 2H), 8.21 (dd, *J* = 8.2, 5.3 Hz, 2H), 8.15 (t, *J* = 7.1 Hz, 1H), 7.67 (dd, *J* = 17.3, 8.3 Hz, 1H), 7.47 (dd, *J* = 12.9, 7.9 Hz, 1H), 6.34 (s, 6H).

#### 3.2.4. Synthesis of [(*η*^6^-Toluene)Ru(L_1_)Cl]Cl (**2**)

Complex **2** was prepared using a similar method, but with [(*η*^6^-toluene)RuCl_2_]_2_ (0.035 mmol, 18.5 mg) and L_1_ (0.07 mmol, 22.54 mg). ESI-MS (in MeOH, *m*/*z*): 558.0227 ([M − Cl]^+^); 1121.0409 ([2M − 2Cl − H]^+^). Calculated for C_27_H_22_ON_4_Cl_4_F_2_Ru (%): C 46.37, H 3.17, N 8.01; Found (%): C 47.48, H 3.09, N 8.02. (One molecule containing1Cl^-^, 1 CH_2_Cl_2_, 1 H_2_O). ^1^H NMR (600 MHz, DMSO-*d*_6_) δ 9.91 (dd, *J* = 5.2, 0.9 Hz, 1H), 9.39 (s, 1H), 8.19 (dt, *J* = 11.6, 5.8 Hz, 1H), 7.68–7.58 (m, 1H), 7.43 (dd, *J* = 12.6, 7.9 Hz, 1H), 6.46 (t, *J* = 6.0 Hz, 1H), 6.10 (d, *J* = 6.2 Hz, 1H), 5.90 (t, *J* = 5.7 Hz, 1H), 5.71 (dd, *J* = 8.9, 5.8 Hz, 1H), 2.29 (s, 1H). ^13^C NMR (151 MHz, DMSO-*d*_6_) δ 154.6 (s), 151.7 (s), 151.7 (s), 150.1 (s), 150.0 (s), 148.8 (s), 148.7 (s), 147.6 (s), 147.1 (s), 147.0 (s), 144.0 (s), 133.2 (s), 126.7 (s), 126.0 (s), 125.8 (s), 120.3 (s), 119.5 (s), 119.4 (s), 106.0 (s), 90.6 (s), 89.9 (s), 85.2 (s), 83.4 (s), 82.6 (s), 80.4 (s), 19.3 (s), 19.0 (s).

#### 3.2.5. Synthesis of [(*η*^6^-*p*-Cymene)Ru(L_1_)Cl]Cl (**3**)

Complex **3** was prepared using a similar method, but with [(*η*^6^-*p*-cymene)RuCl_2_]_2_ (0.035 mmol, 21.4 mg) and L_1_ (0.07 mmol, 22.54 mg). ESI-MS (in MeOH, *m*/*z*): 603.30 ([M − Cl]^+^). Calculated forC_30_H_28_ON_4_Cl_4_F_2_Ru(%): C 48.60, H 3.81, N 7.56; Found (%): C 50.31, H 3.80, N 7.87. (One molecule containing1Cl^-^, 1 CH_2_Cl_2_, 1 H_2_O). ^1^H NMR (600 MHz, DMSO-*d*_6_) δ 9.90 (dd, *J* = 5.3, 0.9 Hz, 2H), 9.43 (s, 2H), 8.22 (dd, *J* = 8.2, 5.3 Hz, 2H), 8.16 (t, *J* = 7.1 Hz, 1H), 7.69–7.58 (m, 1H), 7.45 (dd, *J* = 12.5, 7.9 Hz, 1H), 6.37 (d, *J* = 6.4 Hz, 2H), 6.14 (d, *J* = 6.4 Hz, 2H), 2.62 (dq, *J* = 13.7, 6.9 Hz, 1H), 2.22 (s, 3H), 0.93 (dd, *J* = 21.7, 6.9 Hz, 6H). ^13^C NMR (151 MHz, DMSO-*d*_6_) δ 154.5 (s),151.7 (s), 149.9 (s), 148.7 (s), 147.8 (s), 147.0 (s), 143.8 (s), 133.2 (s), 126.8 (s), 126.1 (s), 125.9 (s), 120.5 (s), 119.4 (s), 119.4 (s), 104.4 (s), 103.5 (s), 86.7 (s), 84.4 (s), 30.9 (s), 22.1 (s), 18.7 (s).

#### 3.2.6. Synthesis of [(*η*^6^-Benzene)Ru(L_2_)Cl]Cl (**4**)

Complex **4** was prepared using a similar method, but with [(*η*^6^-benzene)RuCl_2_]_2_ (0.035 mmol, 17.5 mg) and L_2_ (0.07 mmol, 22.54 mg). ESI-MS (in MeOH, *m*/*z*): 544.00 ([M–Cl]^+^); 1093.00 ([2M − 2Cl − H]^+^). Calculated for C_26_H_20_ON_4_Cl_4_F_2_Ru (%): C 45.57, H 2.94, N 8.18; Found (%):C 46.00, H 3.12, N 8.61. (One molecule containing1Cl^-^, 1 CH_2_Cl_2_, 1 H_2_O). ^1^H NMR (600 MHz, MeOD-*d*_6_) δ 9.86 (d, *J* = 5.2 Hz, 2H), 9.16 (d, *J* = 8.1 Hz, 2H), 8.27 (dd, *J* = 14.9, 8.3 Hz, 1H), 8.08 (dd, *J* = 8.0, 5.3 Hz, 2H), 7.34–7.28 (m, 1H), 7.26 (ddd, *J* = 10.5, 6.8, 2.2 Hz, 1H), 6.28 (s, 6H). ^3^C NMR (151 MHz, MeOD-*d*_6_) δ 165.3 (s), 165.2 (s), 163.6 (s), 163.5 (s), 161.2 (s), 161.1 (s), 159.5 (s), 159.4 (s), 153.8 (s), 147.9 (s), 144.1 (s), 132.5 (s), 131.9 (s), 125.9 (s), 113.9 (s), 112.5 (s), 112.3 (s), 104.6 (s), 104.5 (s), 104.3 (s), 87.0 (s).

#### 3.2.7. Synthesis of [(*η*^6^-Toluene)Ru(L_2_)Cl]Cl (**5**)

Complex **5** was prepared using a similar method, but with [(*η*^6^-toluene)RuCl_2_]_2_ (0.035 mmol, 18.5 mg) and L_2_ (0.07 mmol, 22.54 mg). ESI-MS (in MeOH, *m*/*z*): 559.02 ([M − Cl]^+^); 1121.04 ([2M − 2Cl − H]^+^). Calculated for C_27_H_22_ON_4_Cl_4_F_2_Ru (%): C 46.37, H 3.17, N 8.01; Found (%): C 45.92, H 3.12, N 7.70; (One molecule containing 1Cl^−^, 1 CH_2_Cl_2_, 1 H_2_O). ^1^H NMR (600 MHz, DMSO-*d*_6_) δ 9.90 (dd, *J* = 5.2, 0.9 Hz, 1H), 9.37 (s, 1H), 8.24–8.12 (m, 1H), 7.65–7.53 (m, 1H), 7.45–7.32 (m, 1H), 6.52–6.40 (m, 1H), 6.12 (dd, *J* = 22.9, 6.3 Hz, 1H), 5.93–5.86 (m, 1H), 5.71 (dd, *J* = 9.0, 5.8 Hz, 1H), 2.30 (d, *J* = 11.9 Hz, 2H). ^13^C NMR (151 MHz, DMSO-*d*_6_) δ 164.6 (s), 162.9 (s), 161.0 (s), 159.3 (s), 154.5 (s), 148.0 (s), 143.9 (s), 133.1 (s), 132.6 (s), 126.6 (s), 115.1 (s), 113.2 (s), 113.1 (s), 106.1 (s), 106.0 (s), 105.9 (s), 105.8 (s), 105.6 (s), 90.6 (s), 90.0 (s), 85.2 (s), 83.4 (s), 82.6 (s), 80.4 (s), 19.3 (s), 19.0 (s).

#### 3.2.8. Synthesis of [(*η*^6^-*p*-Cymene)Ru(L_2_)Cl]Cl (**6**)

Complex **6** was prepared using a similar method, but with [(*η*^6^-*p*-cymene)RuCl_2_]_2_ (0.035 mmol, 21.4 mg) and L_2_ (0.07 mmol, 22.54 mg). ESI-MS (in MeOH, *m*/*z*): 603.30 ([M − Cl]^+^). Calculated for C_30_H_32_O_3_N_4_Cl_4_F_2_Ru (%): C 46.34, H 4.15, N 7.21; Found (%): C 46.13, H 4.32, N 7.16. (One molecule containing 1Cl^−^, 1 CH_2_Cl_2_, 3 H_2_O). ^1^H NMR (500 MHz, DMSO-*d*_6_) δ 9.89 (dd, *J* = 5.3, 0.9 Hz, 2H), 9.41 (s, 2H), 8.39 (dd, *J* = 15.3, 8.6 Hz, 1H), 8.21 (dd, *J* = 8.3, 5.3 Hz, 2H), 7.60 (ddd, *J* = 11.4, 9.3, 2.5 Hz, 1H), 7.39 (td, *J* = 8.4, 2.4 Hz, 1H), 6.37 (d, *J* = 6.4 Hz, 2H), 6.13 (d, *J* = 6.4 Hz, 2H), 2.69–2.57 (m, 1H), 2.21 (s, 3H), 0.91 (d, *J* = 6.9 Hz, 6H). ^13^C NMR (126 MHz, DMSO-*d*_6_) δ 164.8 (s), 162.8 (s), 161.1 (s), 159.1 (s), 154.5 (s), 148.1 (s), 143.7 (s), 133.2 (s), 132.6 (s), 126.8 (s), 115.1 (s), 113.2 (s), 113.0 (s), 106.0 (s), 105.7 (s), 105.5 (s), 104.4 (s), 103.5 (s), 86.7 (s), 86.0 (s), 84.4 (s), 30.9 (s), 22.1 (s), 18.7 (s).

## 4. Conclusions

In this study, a series of novel arene Ru(II) complexes with difluorinated ligands were synthesized and characterized. These complexes could bind to and stabilize *c-myc* G-quadruplex DNA. Moreover, the binding of these compounds with some other G-quadruplex DNA, Bcl-2, kras, VEGF, c-kit1 and telomeric may also have occurred, since it is difficult to distinguish different G-quadruplex DNAs that originate from different tumor cells, and this will be investigated in our future studies. Further studies show that these complexes, especially **6**, exhibit promising inhibitory activity against the growth of the U87 cell with an IC_50_ of approximately 23.75 ± 0.61 μM. Studies on the underlying mechanism show that **6** can induce the S-phase arrest of an U87 cell. These arene Ru(II) complexes with difluorinated ligands can be developed as potential stabilizers to *c-myc* G-quadruplex DNA, and could be used to inhibit the growth of glioma cells in clinics.

## Figures and Tables

**Table 1:** Concentrations of DC_50_ values (Ru(II) complexes for the displacement of 50% of the TO from G-quadruplex DNA-TO complexes) for *c-myc* G-quadruplexes as determined from FID assays.

Com.	DC_50_ (μM)					
	1	2	3	4	5	6
*c-myc*	6.6	9.93	10.26	7.6	10.8	15.6

**Table 2:** Anticancer activities of complexes against human cancer cells after 72 h of incubation and the corresponding lipophilicity (mean value of three experiments).

Comp.	IC_50_/μM				
570 nm	MCF-7	A549	HepG2	U87	LogP*o*/*w*
**L_1_**	4.25 ± 0.18	7.99 ± 0.05	0.29 ± 0.01	14.3 ± 0.47	0.375
**L_2_**	1.48 ± 0.09	3.42 ± 0.13	0.12 ± 0.01	5.87 ± 0.27	0.218
**1**	>100	95.03 ± 2.44	>100	>100	−1.145
**2**	>100	92.58 ± 5.34	>100	>100	−1.251
**3**	>100	41.58 ± 1.80	>100	51.61 ± 1.64	−0.6615
**4**	>100	81.35 ± 4.96	>100	>100	−1.414
**5**	>100	80.47 ± 2.56	>100	>100	−1.428
**6**	>100	73.20 ± 0.63	>100	23.75 ± 0.61	−0.8077
Cisplatin	15.27 ± 0.18	17.27 ± 0.26	9.90 ± 0.06	32.59 ± 0.85	-

## Data Availability

Dates of the compounds are available from the authors.

## Supplementary Materials

The following supporting information can be downloaded at: https://www.mdpi.com/article/10.3390/molecules27061897/s1, Figure S1: ESI-MS spectra of ligand L1, Figure S2: ESI-MS spectra of ligand L2, Figure S3: ESI-MS spectra of complex 1, Figure S4: ESI-MS spectra of complex 2, Figure S5: ESI-MS spectra of complex 3, Figure S6: ESI-MS spectra of complex 4, Figure S7: ESI-MS spectra of complex 5, Figure S8: ESI-MS spectra of complex 6, Figure S9: 1H-NMR and 13C-NMR spectra of ligand L1, Figure S10: 1H-NMR and 13C-NMR spectra of ligand L2, Figure S11: 1H-NMR spectra of complex 1, Figure S12:1H-NMR and 13C-NMR spectra of complex 2, Figure S13: 1H-NMR and 13C-NMR spectra of complex 3, Figure S14: 1H-NMR and 13C-NMR spectra of complex 4, Figure S15: 1H-NMR and 13C-NMR spectra of complex 5, Figure S16: 1H-NMR spectra of Ru(II) complexes 6, Figure S17: Effect of 6 on the PCR-stop assay with c-myc G4 DNA, Figure S18: The stability of complex 6 in a Tris buffer solution, Figure S19: Ligand(L2)-c myc G4 DNA interactions, Table S1: The cytotoxic activity of the ligands and arene (II)-modified compounds, Figure S20: Multiple gene expression in glioblastoma multiforme, Figure S21: Binding site and mode of the arene Ru(II) complexes that interacted with c-myc G-quadruplex DNA analyzed by molecular docking; Figure S22: The UV–vis absorption titrations of arene Ru(II) complexes modified with and without F atom.

## Sample Availability

Samples of the compounds are available from the authors.
