# Supplementary material for: Arene Ru(II) Complexes with Difluorinated Ligands Act as Potential Inducers of S-Phase Arrest via the Stabilization of c-myc G-Quadruplex DNA
Source: Molecules. 2022 Mar 15;27(6):1897. doi: 10.3390/molecules27061897 (PMC8954944; doi:10.3390/molecules27061897)
Supplement: Supplementary file 1 [file molecules-27-01897-s001.zip › molecules-1587993-supplementary.pdf]

Supporting Information
for

Arene Ru(II) complexes with difluorinated ligands acted as potential inducer of S-phase arrest via stabilizing *c-myc* G-quadruplex DNA

Liang Zeng [1,#], Chanling Yuan [2,#], Jing Shu [2], Jiayi Qian [2], Qiong Wu [4], Yanhua Chen [2], Ruzhen Wu [2], Xiaoming Ou yang [3,*], Yuan Li [1], and Wenjie Mei [2,4,*]

[1]Department of Pathology, Guangzhou Women and Children's Medical Center, Guangzhou Medical University

[2]School of Pharmacy, Guangdong Pharmaceutical University; yuanban@gdpu.edu.cn

[3]Department of pathology, The Second Affiliated Hospital of Guangzhou Medical University

[4]Guangdong Province Engineering and Technology Centre for Molecular Probe and Biomedicine Imaging

*Correspondence: wenjiemei@126.com; gzoyxm@163.com;

Contents:

1. ESI–MS spectra of ruthenium(II) complexes (Figure S1- S8)	pages 1-4
2. ^{1}H-NMR and^{13}C-NMR spectra of complexes (Figure S9-S16)	pages 5-12
3. Effect of **6** on the PCR-stop assay with *c-myc* G4 DNA(Figure S17)	pages 12
4. The stability of complex **6** in Tris buffer solution(Figure S18)	pages 13
5. Ligand(L_2)-*c myc* G4 DNA interactions(Figure S19)	pages 13
6. The cytotoxic activity of the ligands and arene Ru(II)-modified compounds(Table S1)	pages 14
7. Multiple gene expression in glioblastoma multiforme(Figure S20)	pages 14
8. Binding site and mode of the arene Ru(II) complexes interacted with c-myc G-quadruplex DNA analyzed by molecular docking(Figure S21)	pages 14-15
9. The UV–vis absorption titrations of arene Ru(II) complexes modified with and without F atom(Figure S22)	pages 16

1. ESI–MS spectra of ruthenium(II) complexes

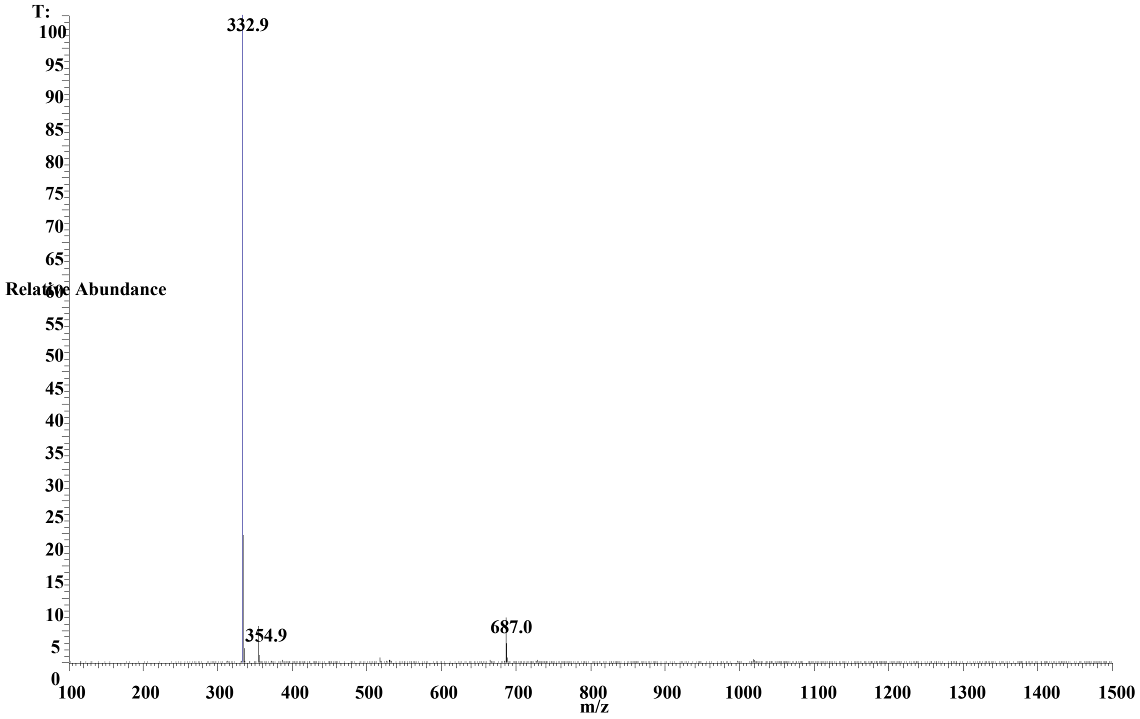

Figure S1. ESI-MS spectra of ligand $\mathbf{L_1}$.

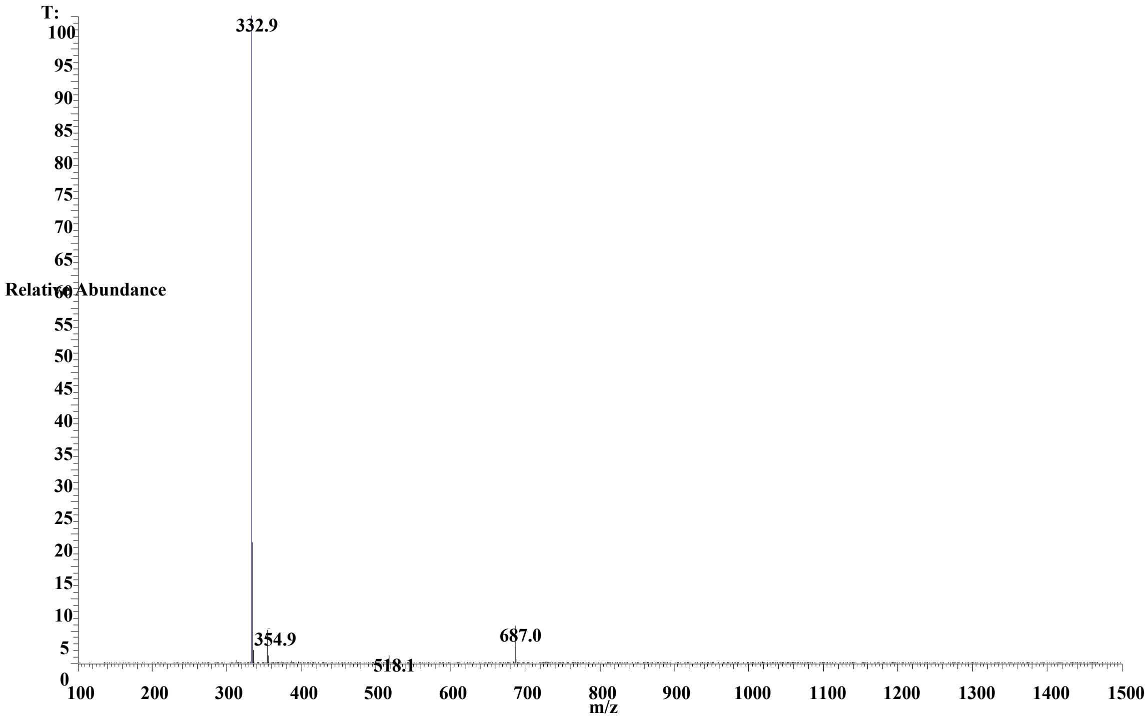

Figure S2. ESI-MS spectra of ligand $\mathbf{L_2}$.

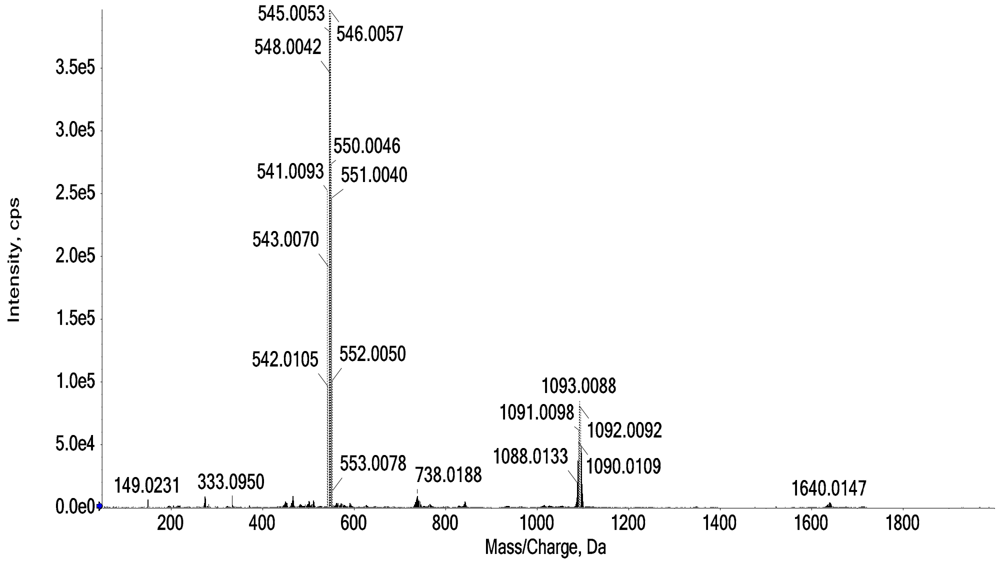

Figure S3. ESI-MS spectra of complex **1.**

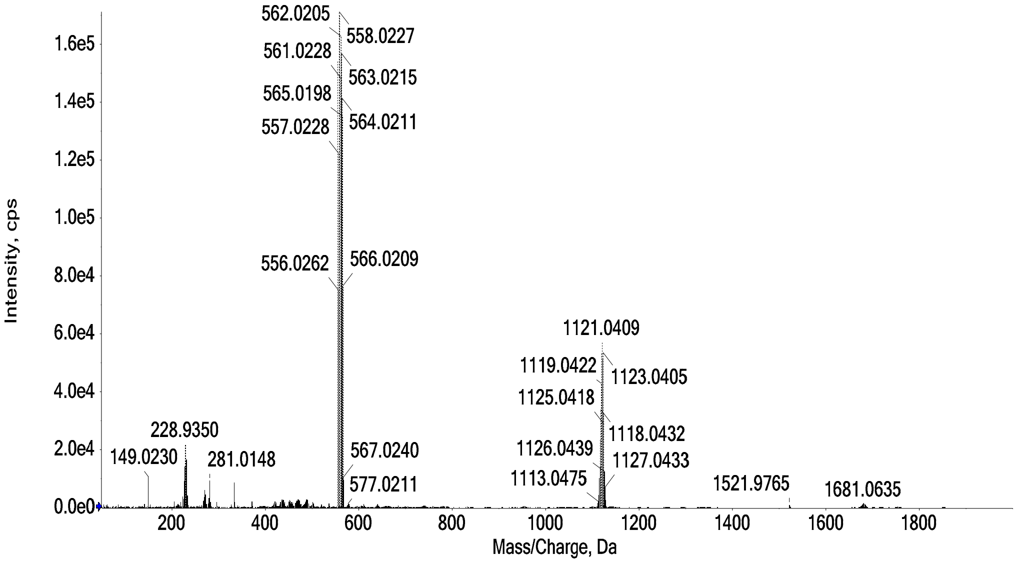

Figure S4. ESI-MS spectra of complex **2.**

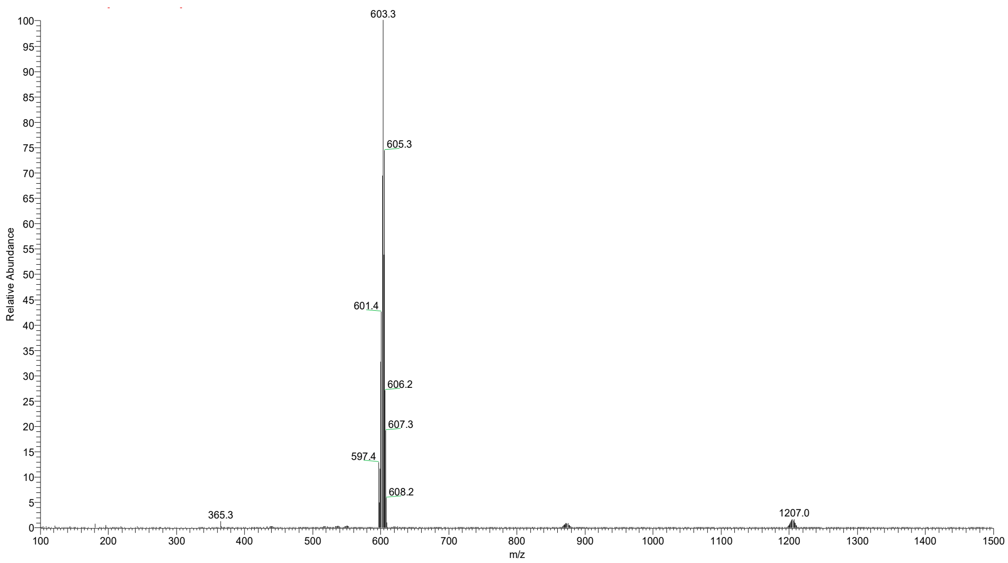

Figure S5. ESI-MS spectra of complex **3.**

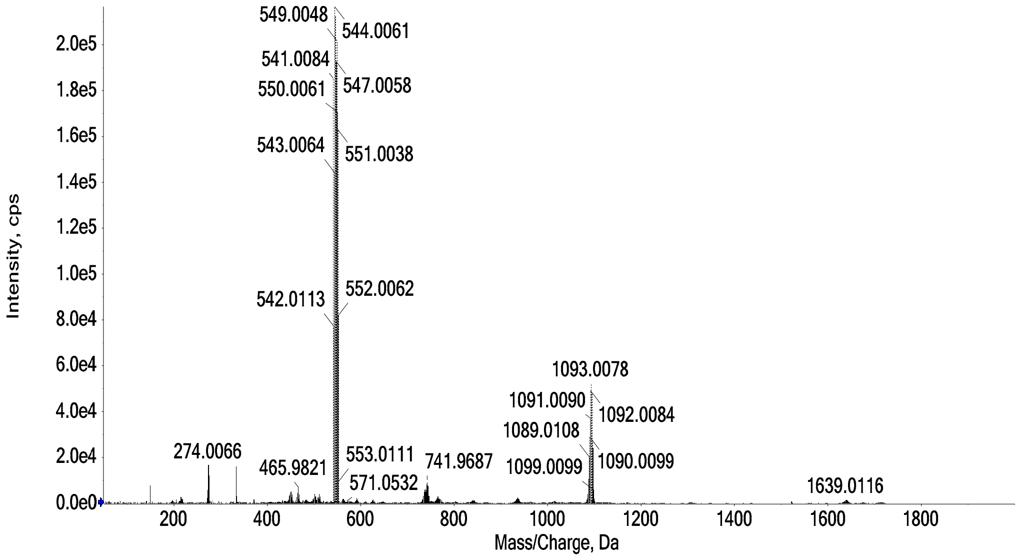

Figure S6. ESI-MS spectra of complex **4.**

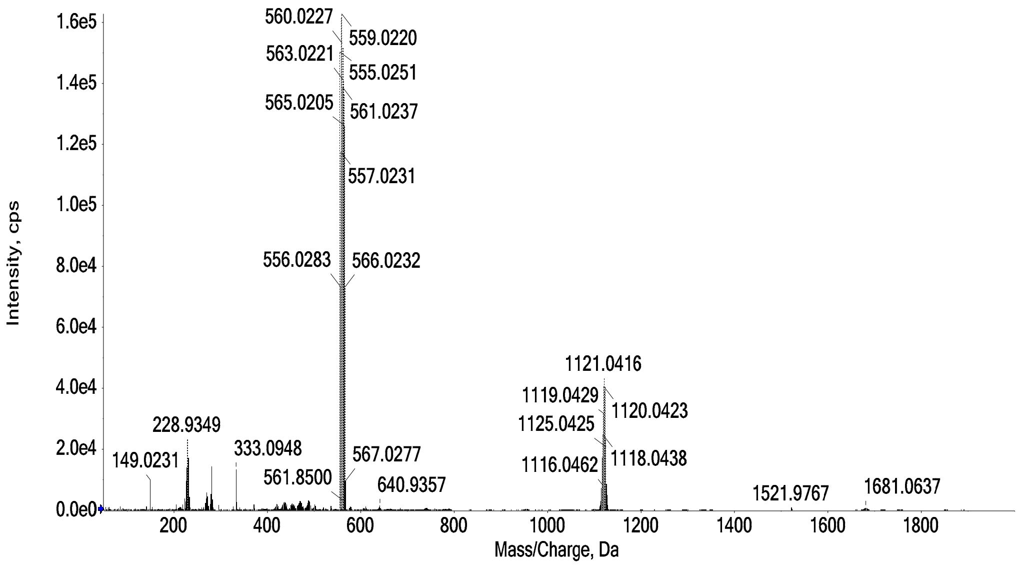

Figure S7. ESI-MS spectra of complex **5.**

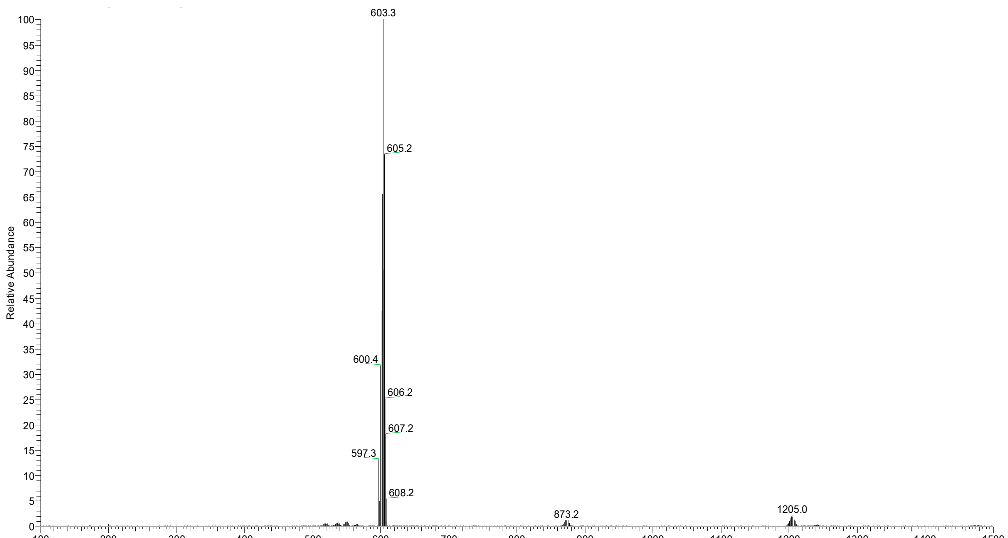

Figure S8. ESI-MS spectra of complex **6.**

2. ^{1}H-NMR and ^{13}C-NMR spectra of complexes

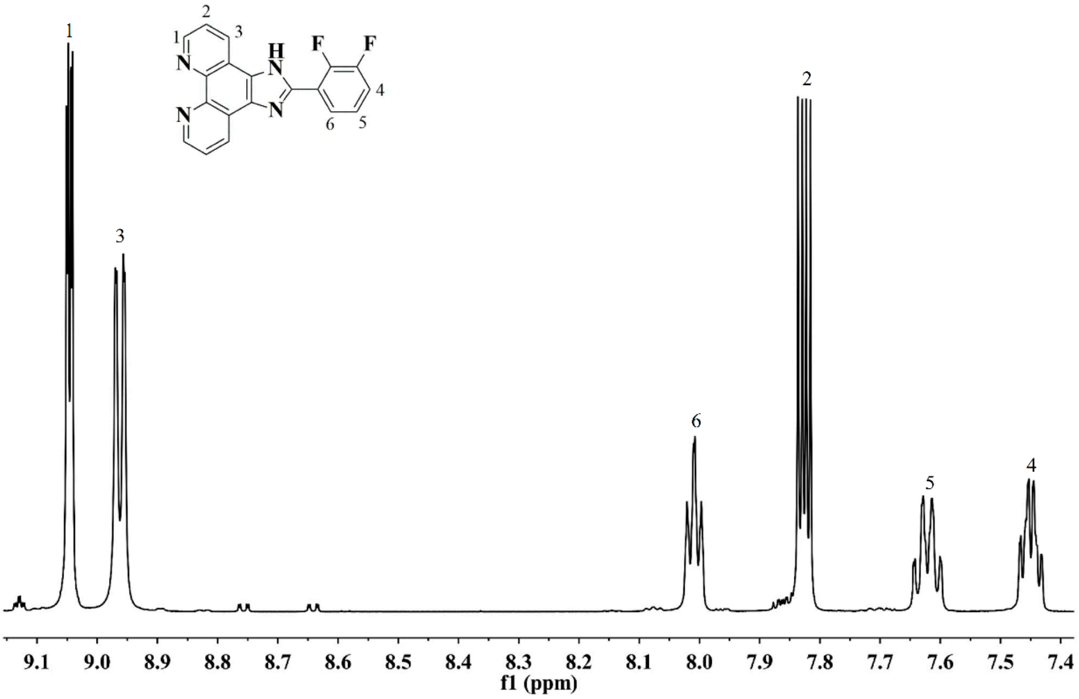

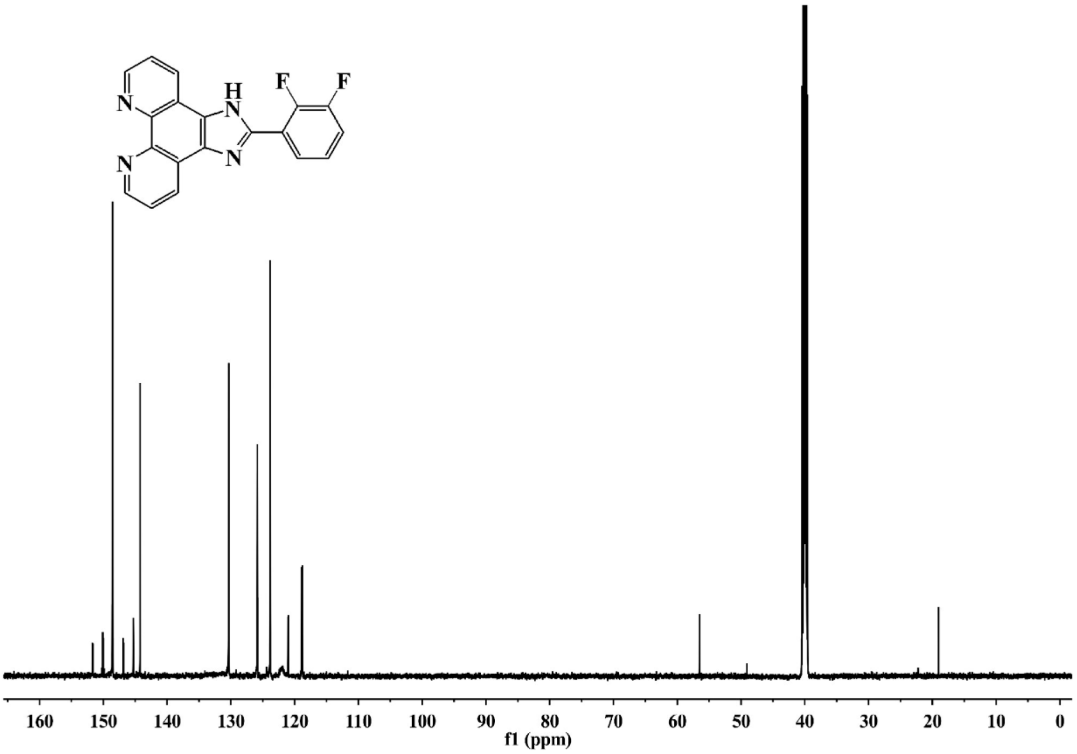

Figure S9. ^{1}H-NMR and ^{13}C-NMR spectra of ligand $\mathbf{L_1}$.

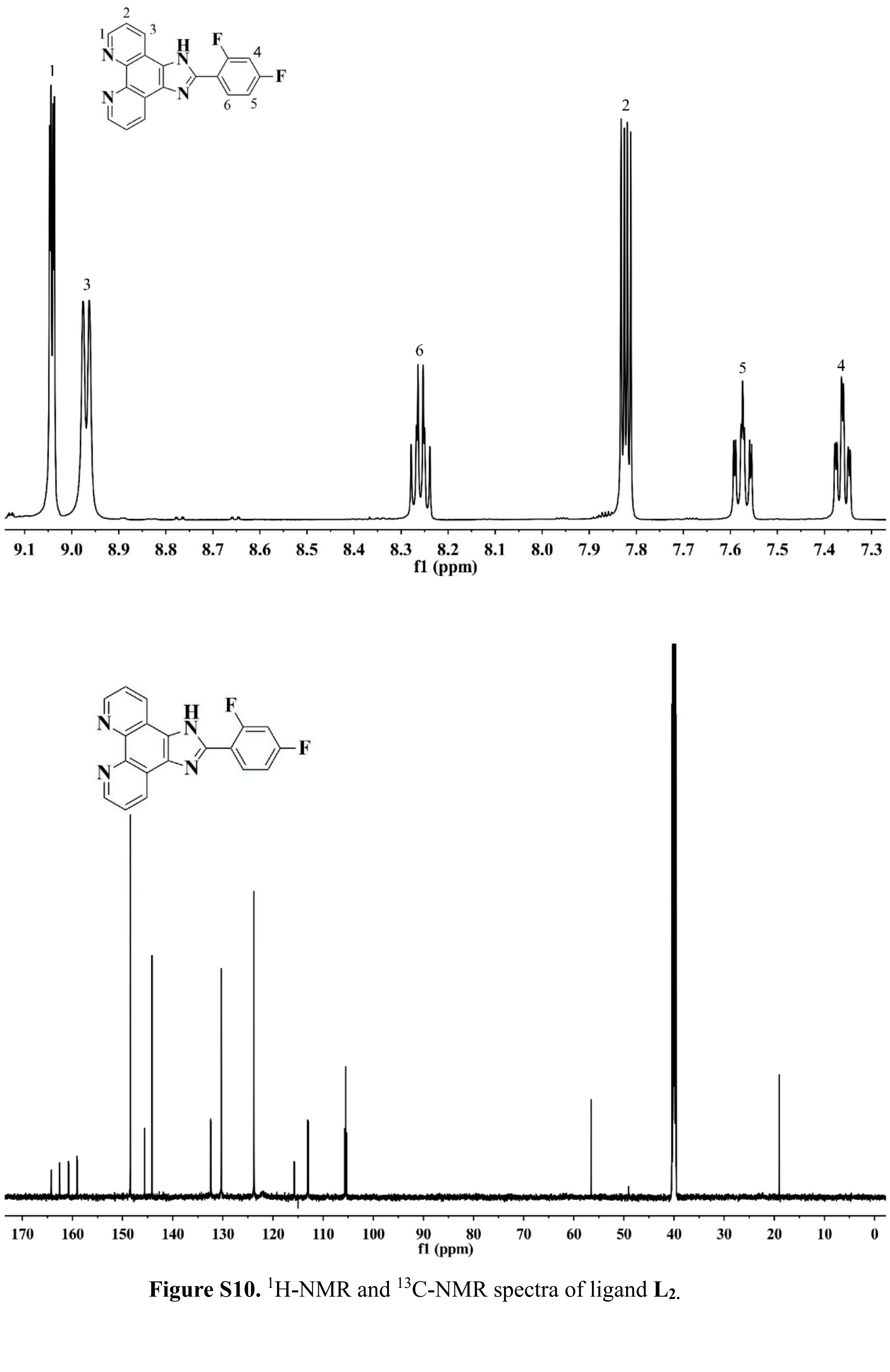

Figure S10. ^{1}H-NMR and ^{13}C-NMR spectra of ligand $\mathbf{L_2}$.

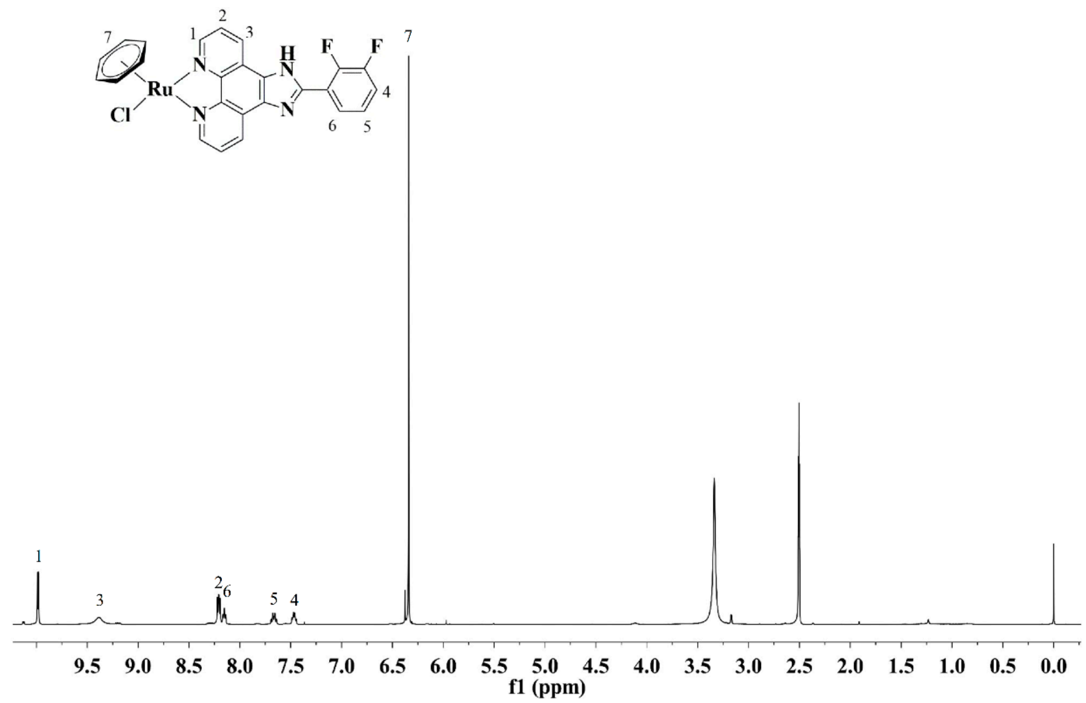

Figure S11. ^{1}H-NMR spectra of complex **1**.

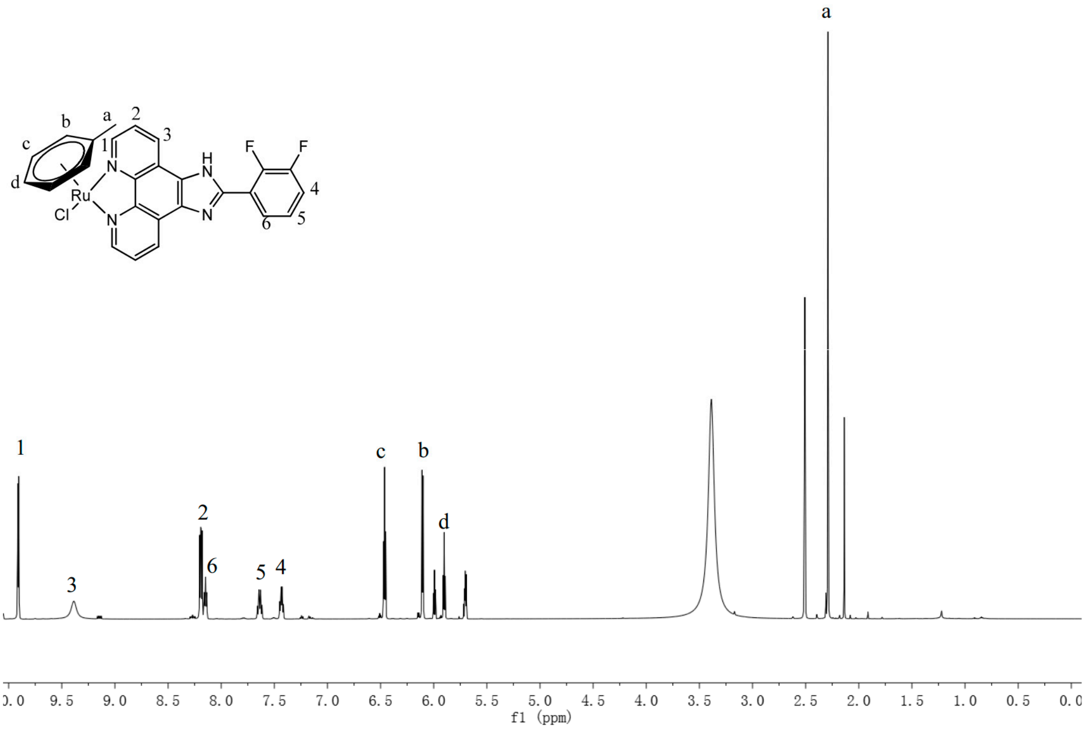

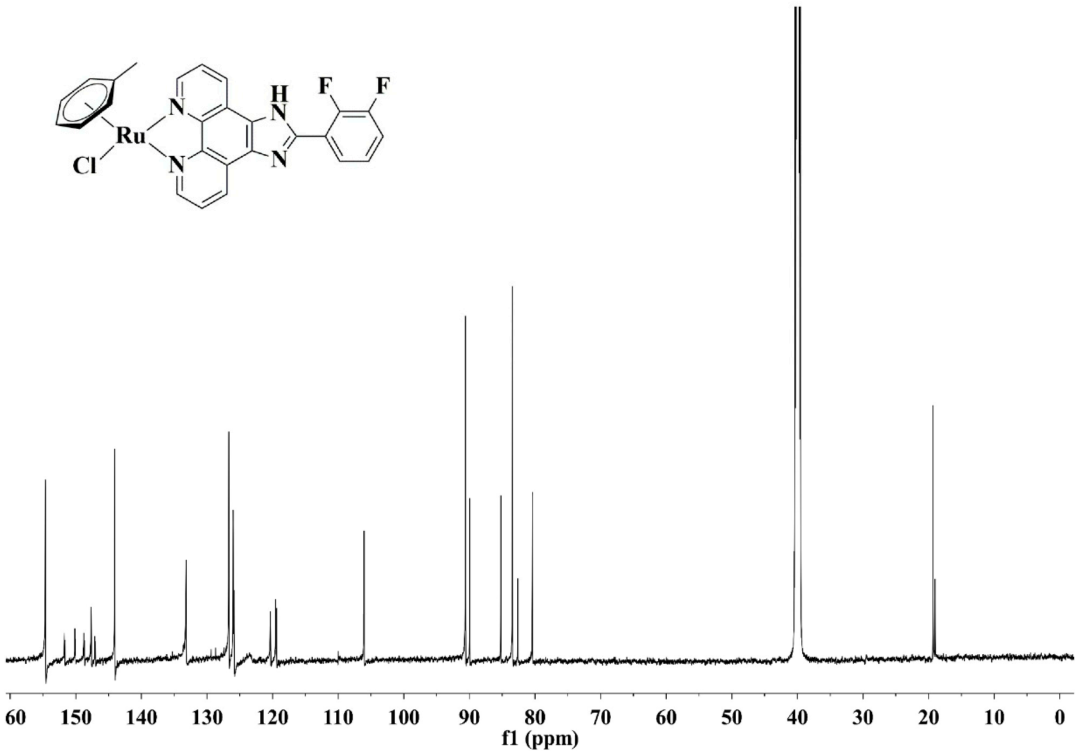

Figure S12. ¹H-NMR and ¹³C-NMR spectra of complex **2.**

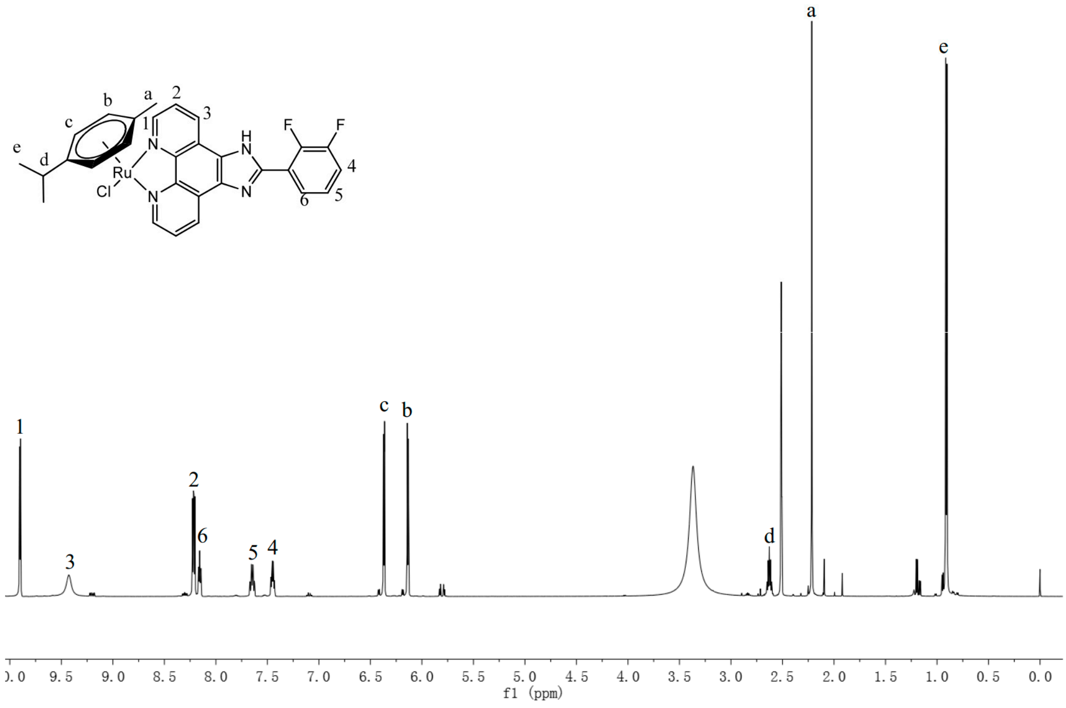

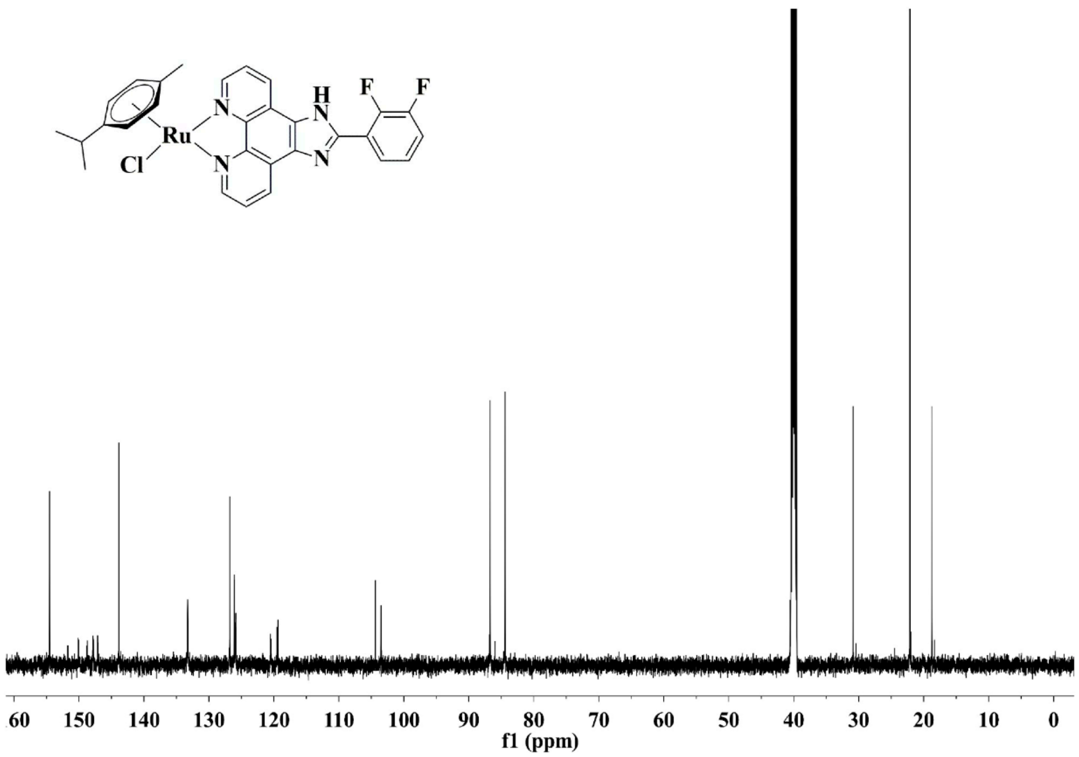

Figure S13. ^{1}H-NMR and ^{13}C-NMR spectra of complex **3.**

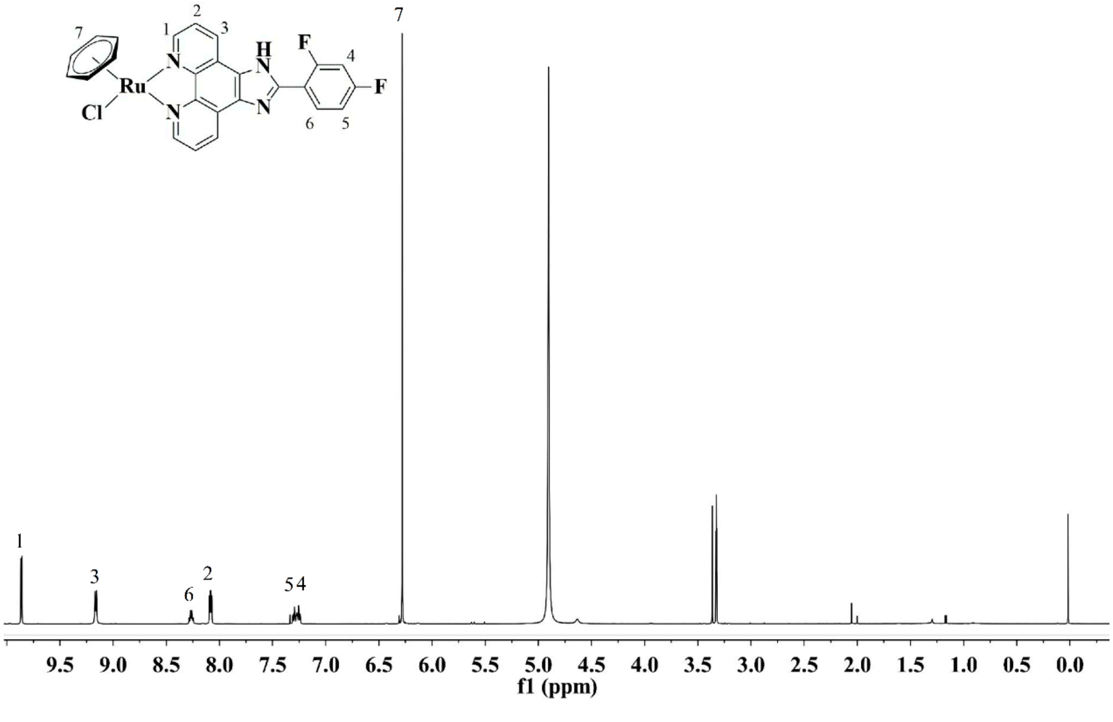

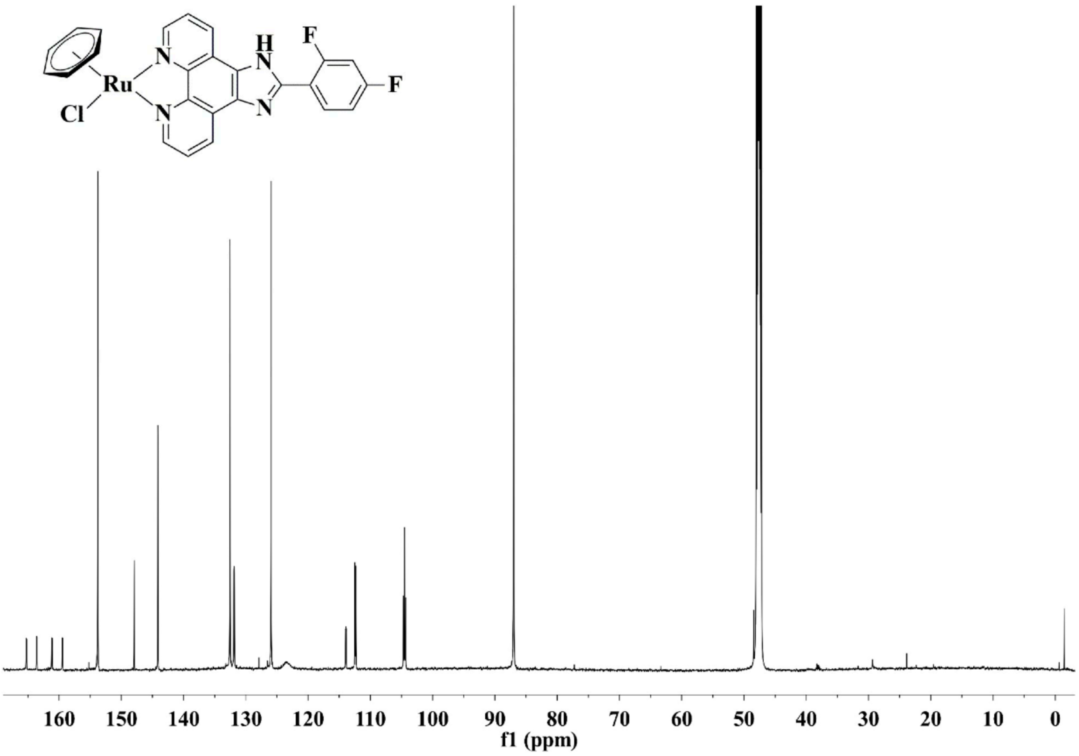

Figure S14. ^{1}H-NMR and ^{13}C-NMR spectra of complex **4.**

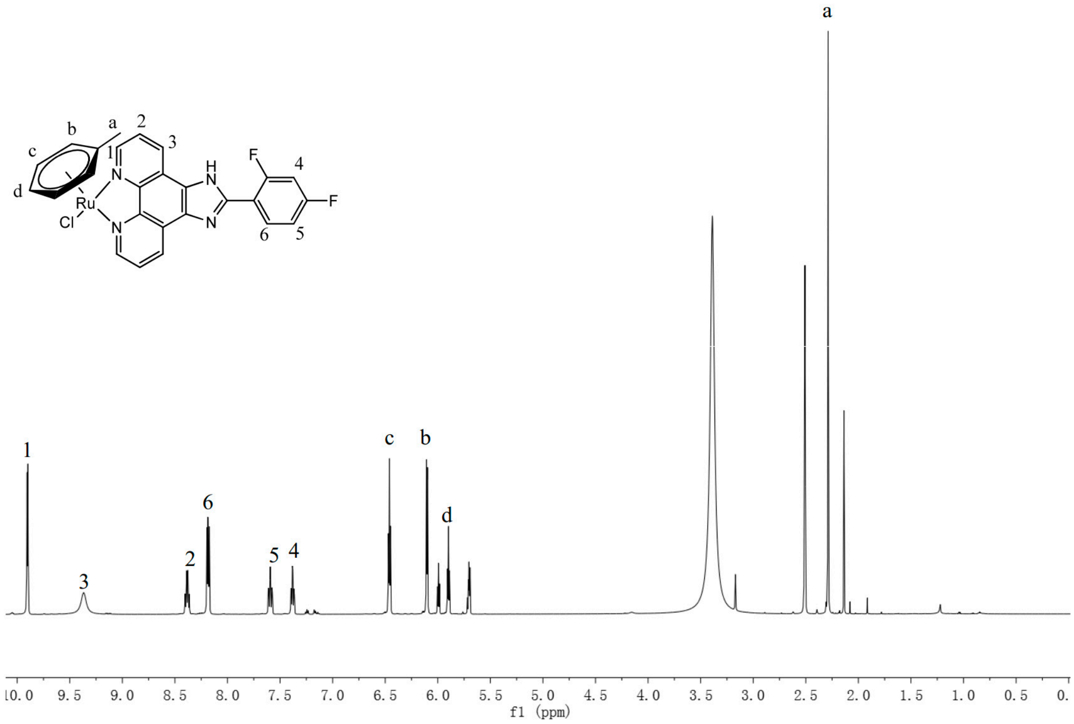

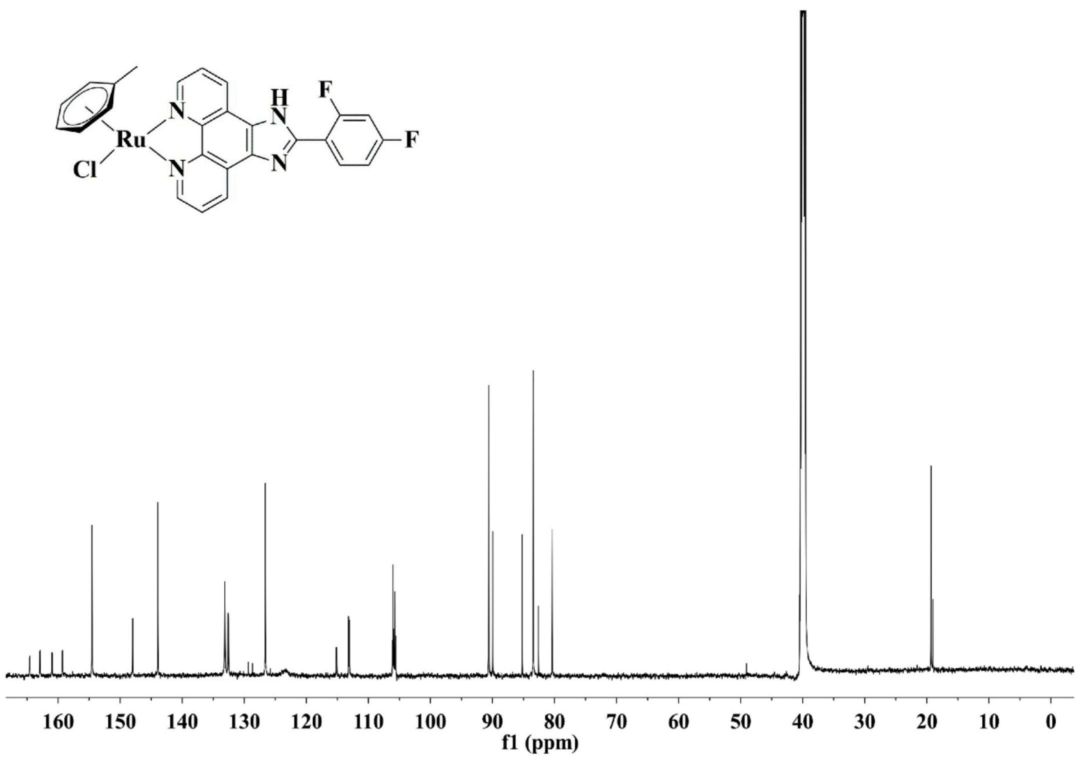

Figure S15. ^{1}H-NMR and ^{13}C-NMR spectra of complex **5.**

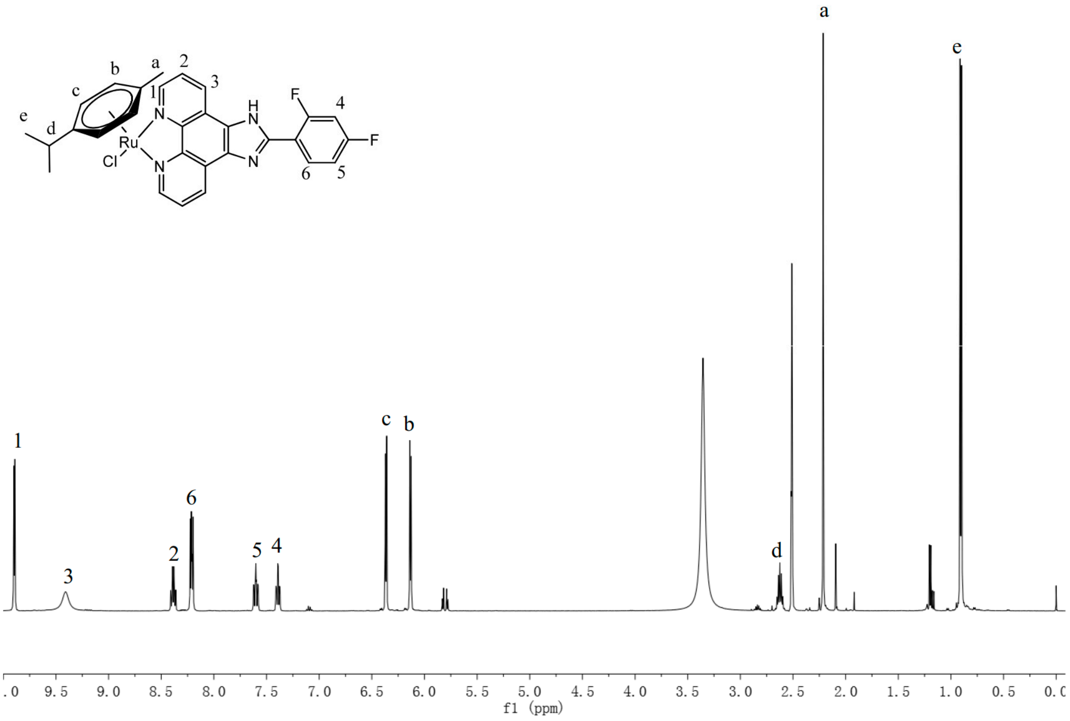

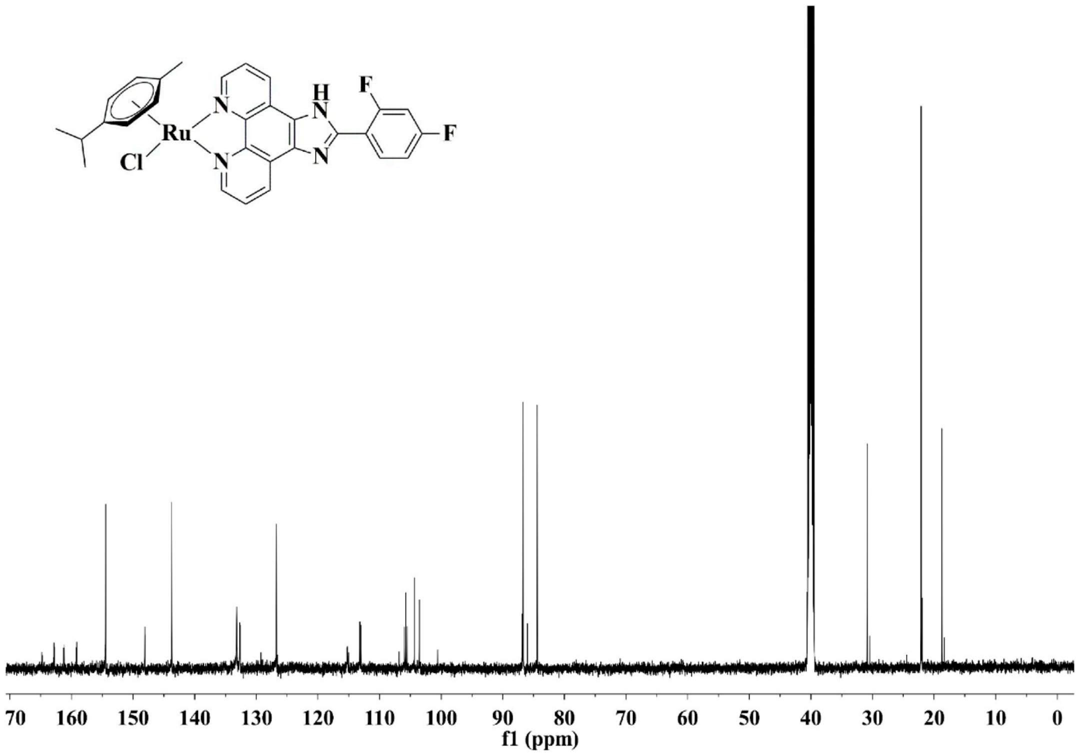

Figure S16. ^{1}H-NMR and ^{13}C-NMR spectra of complex **6**.

3. Effect of 6 on the PCR-stop assay with *c-myc* G4 DNA

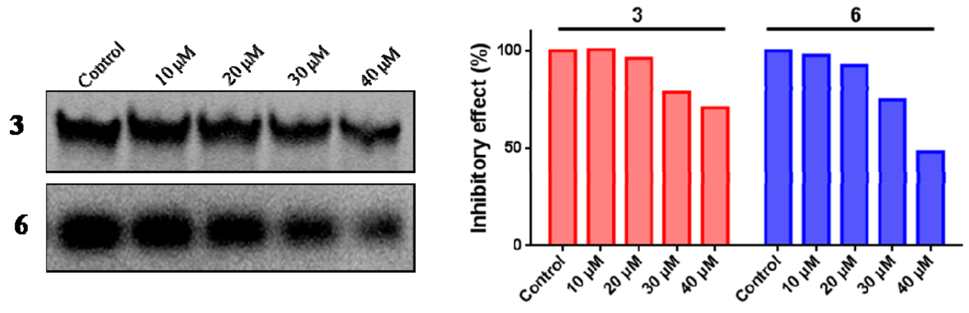

A B

Figure S17. (A) The inhibitory effect of complexes **3** and **6** on the PCR-stop assay with *c-myc* G-quadruplex DNA. [Ru] = 0, 10, 20, 30 and 40 μM, [*c-myc*] = 10 pM. (B) The replication blocking of PCR products obtained for different complex concentrations.

4. The stability of complex 6 in Tris buffer solution

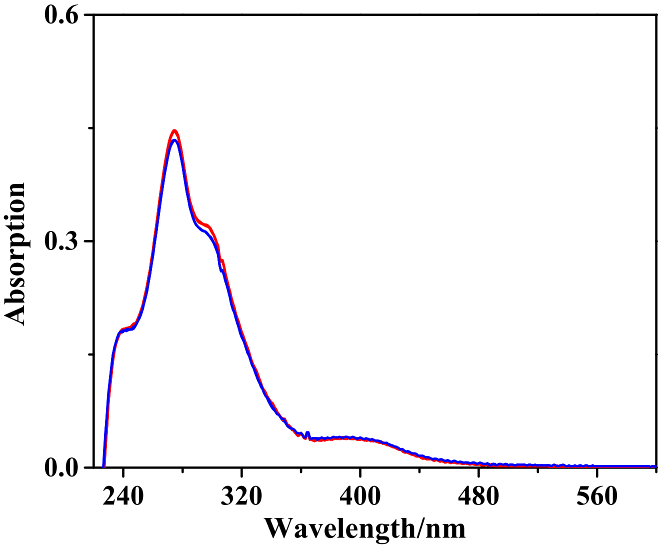

Figure S18. The stability of **6** in Tris butffer solution. The red line is the data from day one, and the blue line is the data from day three. The variation value of absorption peak is 2.7%. The experimental results showed that the UV-vis spectra of the compound after three days in the buffer solution were consistent with that of the first day of preparation. Deviation but permissible.

5. Ligand(L_2)-*c myc* G4 DNA interactions

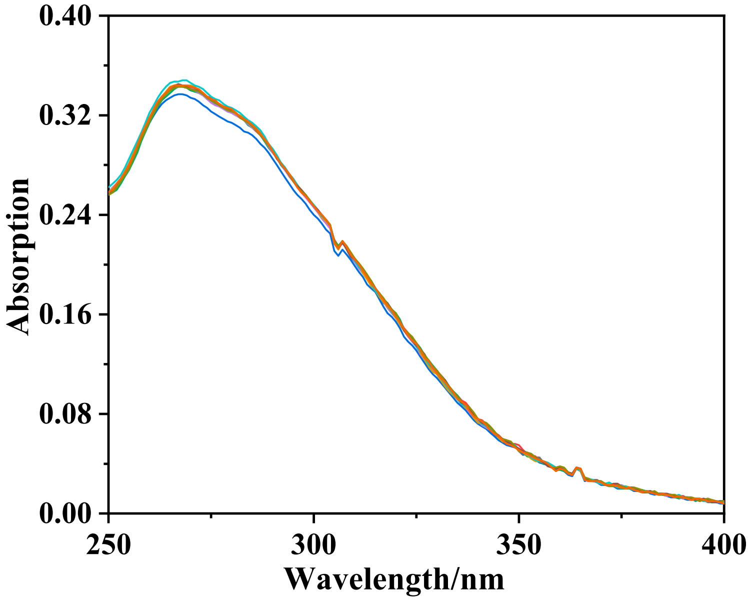

Figure S19. Ligand(**L_2**)-*c myc* G4 DNA interactions. UV-visible spectra of 2-(2,4-di-fluorophenyl) imidazole[4,5f] [1,10]-phenanthroline **(L_2)** in 10 mM Tris-HCl buffer (pH 7.4) containing 100 mM KCl. [DNA]=100 μM. The UV absorption peak of the ligand **L_2** did not change significantly after the addition of DNA. Compared with the difluorine modified compounds, the binding ability of the ligand to DNA was poor.

6. The cytotoxic activity of the ligands and arene Ru(II)-modified compounds

Table S1. The cytotoxic activities of complexes against human keratinocyte Hacat cells after 72 h incubation.

Comp.	$\mathbf{L_1}$	$\mathbf{L_2}$	**3**	**6**
IC50/μM	1.85 ± 0.03	2.72 ± 0.06	68.15 ± 1.77	79.32 ± 0.83

7. Multiple gene expression in glioblastoma multiforme

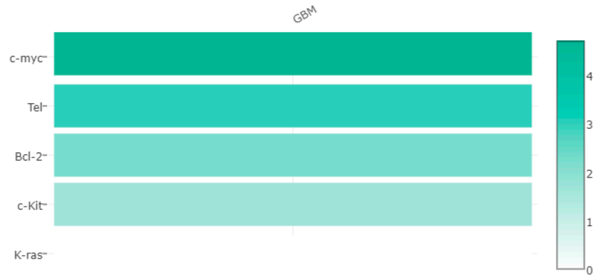

Figure S20. Multiple gene expression in glioblastoma multiforme(Copyright © 2017 Zefang Tang, Chenwei Li, Boxi Kang. Zhang's Lab.). The darker the color, the higher the gene expression in glioblastoma multiforme. As shown in the heat map, *c-myc* gene is highly expressed in glioblastoma multiforme (GBM) compared with other G-quadruplex DNA (*Tel, Bcl-2, c-kit, k-ras*).

8. Binding site and mode of the arene Ru(II) complexes interacted with *c-myc* G-quadruplex DNA analyzed by molecular docking

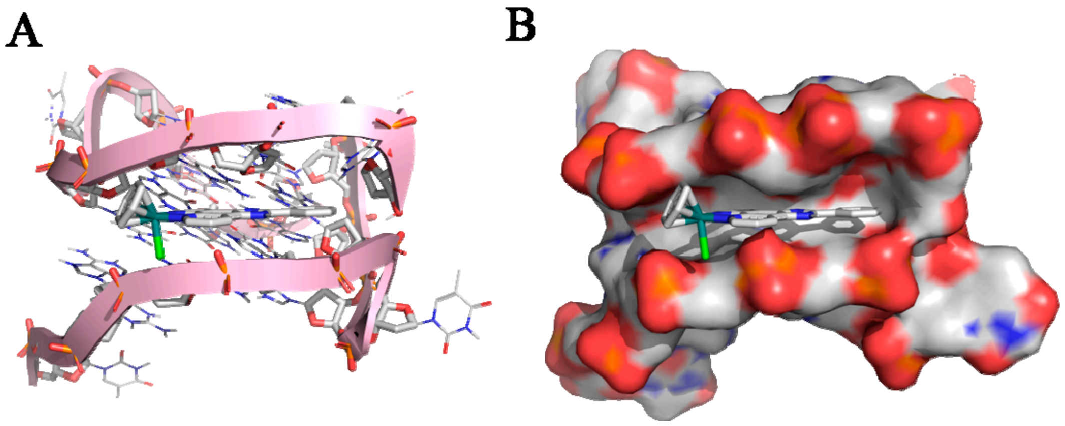

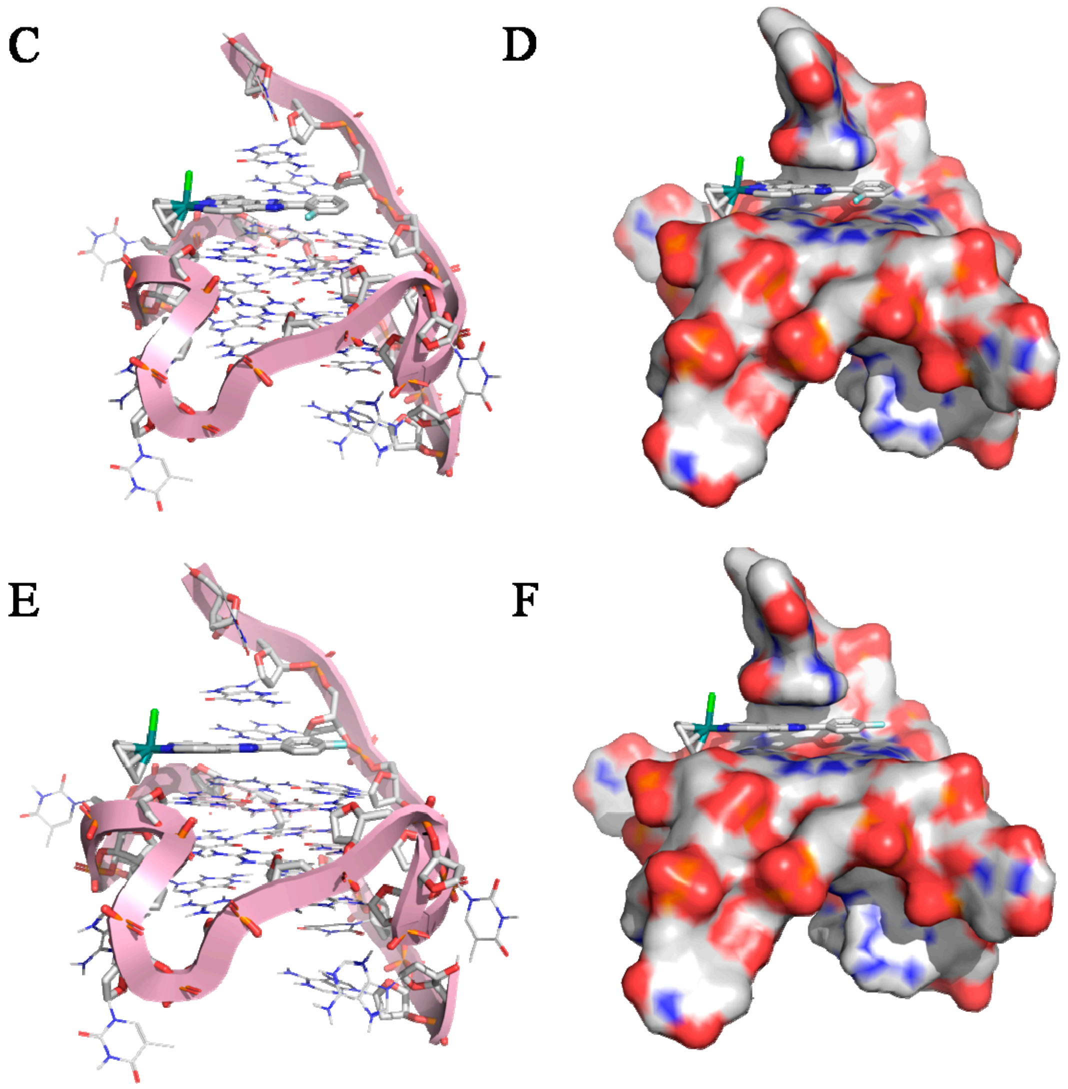

Figure S21. Binding site and mode of the arene Ru(II) complexes interacted with *c-myc* G-quadruplex DNA analyzed by molecular docking. Right (G-quadruplex is rendered with hydrophobic surface of molecular structure), Left (G-quartets are displayed in a stick mode) (A) without F atom, (B) F atom at ortho-position, (C) F atom at ortho-position.

9. The UV–vis absorption titrations of arene Ru(II) complexes modified with and without F atom.

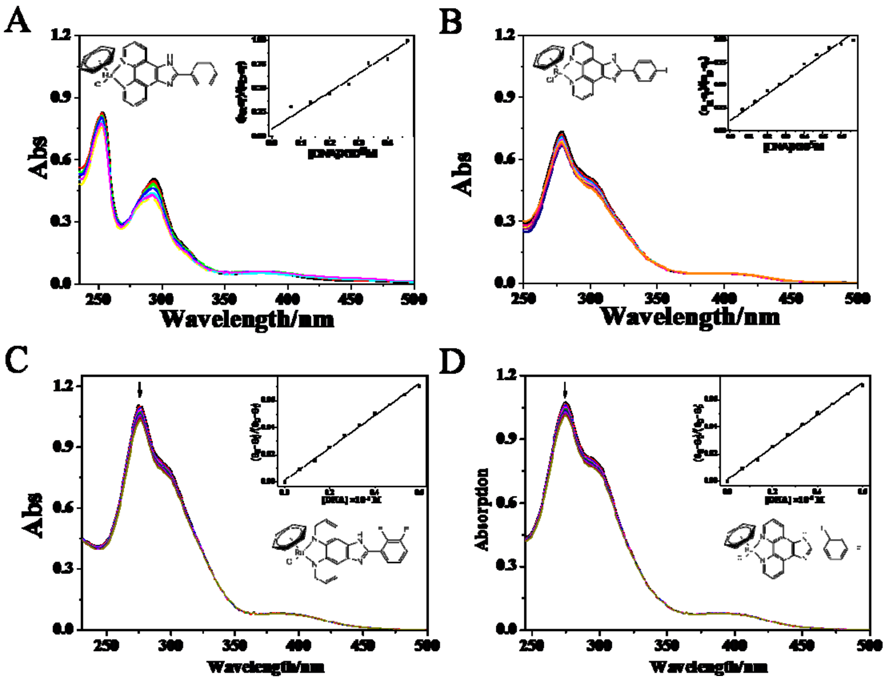

Figure S22. The UV–vis absorption titrations of arene Ru(II) complexes modified with and without F atom at concentration of 20 μM with the increasing of *c-myc* in the Tris–HCl–KCl buffer.
